# A novel approach for designing efficient broadband photodetectors expanding from deep ultraviolet to near infrared

**DOI:** 10.1038/s41377-022-00777-w

**Published:** 2022-04-11

**Authors:** Nan Ding, Yanjie Wu, Wen Xu, Jiekai Lyu, Yue Wang, Lu Zi, Long Shao, Rui Sun, Nan Wang, Sen Liu, Donglei Zhou, Xue Bai, Ji Zhou, Hongwei Song

**Affiliations:** 1 State Key Laboratory on Integrated Optoelectronics, College of Electronic Science and Engineering, Jilin University, Changchun, 130012 China; 2 Key Laboratory of New Energy and Rare Earth Resource Utilization of State Ethnic Affairs Commission, Dalian Minzu University, Dalian, 116600 China; 3 State Kay Lab of New Ceramics and Fine Processing, Department of Materials Science and Engineering, Tsinghua University, Beijing, 100084 China

**Keywords:** Quantum dots, Optoelectronic devices and components

## Abstract

Broadband photodetection (PD) covering the deep ultraviolet to near-infrared (200–1000 nm) range is significant and desirable for various optoelectronic designs. Herein, we employ ultraviolet (UV) luminescent concentrators (LC), iodine-based perovskite quantum dots (PQDs), and organic bulk heterojunction (BHJ) as the UV, visible, and near-infrared (NIR) photosensitive layers, respectively, to construct a broadband heterojunction PD. Firstly, experimental and theoretical results reveal that optoelectronic properties and stability of CsPbI_3_ PQDs are significantly improved through Er^3+^ doping, owing to the reduced defect density, improved charge mobility, increased formation energy, tolerance factor, etc. The narrow bandgap of CsPbI_3_:Er^3+^ PQDs serves as a visible photosensitive layer of PD. Secondly, considering the matchable energy bandgap, the BHJ (BTP-4Cl: PBDB-TF) is selected as to NIR absorption layer to fabricate the hybrid structure with CsPbI_3_:Er^3+^ PQDs. Thirdly, UV LC converts the UV light (200–400 nm) to visible light (400–700 nm), which is further absorbed by CsPbI_3_:Er^3+^ PQDs. In contrast with other perovskites PDs and commercial Si PDs, our PD presents a relatively wide response range and high detectivity especially in UV and NIR regions (two orders of magnitude increase that of commercial Si PDs). Furthermore, the PD also demonstrates significantly enhanced air- and UV- stability, and the photocurrent of the device maintains 81.5% of the original one after 5000 cycles. This work highlights a new attempt for designing broadband PDs, which has application potential in optoelectronic devices.

## Introduction

Photodetectors (PDs) are the technical functional components for capturing and converting ultraviolet (UV) to near-infrared (NIR) photons into electronic outputs^1–5^. The broadband optical detection ability, especially from UV to NIR range, is critical for applications including medical monitoring, video imaging, optical communication, and civil engineering^6–12^. Generally, the commercial silicon PDs present the relatively broad wavelength response range from 400–1100 nm^13,14^, but usually suffer from high cost and low detectivity, especially in the UV region. Solution-processable broadband PDs based on soluble materials have numerous advantages of low cost, simple preparation, and high sensitivity, which has become the next generation of new detectors^15–17^.

Encouragingly, solution-processable metal halide perovskites process outstanding characteristics of large absorption coefficient, long diffusion length, low trapping density, and high photoluminescent quantum efficiency (PLQY), which have shown unprecedented radical progress for various optoelectronic devices, including solar cells (SCs), light-emitting diodes (LEDs), and photodetectors (PDs)^11,18,19^. Among them, all-inorganic perovskite quantum dots (ABX_3_, A = Cs; B = Pb, Ge, Sn; X = Cl, Br, I) (PQDs) have attracted extensive interest in broadband PDs, owing to their wide-range tunability of bandgap, large absorption cross-section, high carrier mobility, etc^18,20–23^. Especially, CsPbI_3_ PQDs process narrow bandgap of 1.73 eV, becoming a candidate for broadband PDs^24^. For example, Tian et al. fabricated 2-aminoethanethiol (AET)/CsPbI_3_ PQDs composite-based PDs device, exhibiting a high responsivity of 105 mA W^−1^ and the detection wavelength covering the visible light^22^. However, its spectrum covers mainly the blue to visible light range (400–700 nm), short of UV response and NIR absorption, due to the insensitivity to UV light and limitation of the bandgap. In addition, they also encounter relatively high trap density, poor carrier mobility, and high susceptibility to moisture and UV light, generating phase transition from cubic to orthorhombic phase^25–27^. The above issues severely limit its photodetection of broadband response spectrum with high stability and responsivity.

To overcome the challenges mentioned above, much efforts have been made to improve the stability and responsivity, and to expand the spectral response range of perovskite-based PDs. A number of metal ions (eg., Zn^2+^, Cr^3+^, Nd^3+^, Er^3+^, Ce^3+^) doping have been proved to be a promising way to boost the optical and electrical performance of perovskite materials^28–31^, including the decrease of trap density and the improvements of carrier mobility, stability, and photoluminescence quantum yield (PLQY). Meanwhile, the strategy of integrating perovskite with NIR absorption materials (e.g., organic bulk heterojunction (BHJ), lead sulfide quantum dots, etc.) was attempted to expand the spectral response range of PDs to the NIR region^32–34^. For example, Chen et al. achieved broadband photodetectors with high NIR external quantum efficiency of over 70% in organic-inorganic perovskite/BHJ hybrid^35^. Nevertheless, such PD has low responsivity in the UV region and relatively poor stability of organic-inorganic perovskite.

The scheme of luminescent conversion was proven to be an effective route to enlarge the response to the UV by absorbing and converting UV to visible photons and further being captured by PD. The luminescent conversion films consisting of Cr^3+^, Ce^3+^, Yb^3+^ tridoped CsPbCl_3_ PQDs or carbon dots were explored to boost the UV response of silicon PDs^36–38^. As a class of excellent luminescent conversion materials, luminescent concentrator (LC) consists of transparent polymer sheets doped with luminescent species that can be employed as a nonimaging optical device that collects and concentrates light energy^39,40^. It has been widely applied in photovoltaic cells or optical communications to largely improve the power conversion efficiency^41,42^.

In this work, we represent the design and fabrication of a novel type of hybrid PDs based on ultraviolet (UV) luminescent concentrators (LC) and doped PQDs and an organic bulk heterojunction, which can realize efficient photodetection in the whole range of 200–1000 nm. The device integrates a tridoped PQDs (CsPbCl_3_:Cr^3+^,Ce^3+^,Mn^2+^) photoluminescent layer to harvest and converts UV light to visible, a CsPbI_3_:Er^3+^ layer to realize the photoelectric conversion of visible light, and an organic bulk heterojunction to extend photoelectric response to NIR light. In such a device, CsPI_3_:Er^3+^ PQDs was explored as the visible photoelectric layer. Doping of Er^3+^ largely improved the radiative transition rate of the perovskite excitons and structure stability, altered charge carrier transport of CsPbI_3_ QDs, thus leading to performance enhancement considerably. In CsPbCl_3_:Cr^3+^,Ce^3+^,Mn^2+^ based LC, Mn^2+^ ions convert the UV to red lights, locating within the optimum regions of CsPbI_3_:Er^3+^ based PD, and simultaneously, Cr^3+^ and Ce^3+^ doping significantly improve PLQY of the PQDs and enhance light-harvesting of UV light for PQDs due to the coupling of *5d* states of Ce^3+^ with the PQDs, extremely in the deep UV (DUV) region. In addition, an organic BHJ (BTP-4Cl: PBDB-TF) with an absorption extending 1000 nm was adopted as NIR photoelectric layer to integrate with CsPbI_3_:Er^3+^ layer. Taking all advantages above, the present PDs realize the spectral response spanning from 200 to 1000 nm and demonstrated detectivity reaching 10^12^ Jones.

## Results

**Fig. 1:**
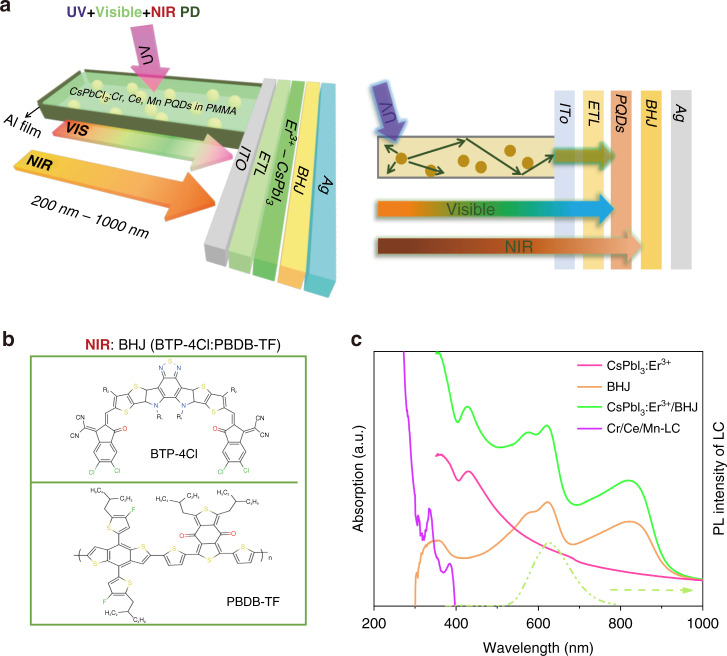
Broadband photodetection based on Cr/Ce/Mn-LC and BHJ and CsPbI_3_:Er^3+^ PQDs. **a** Schematic device structure and 2D view of a broadband PD. **b** The molecule structures of BTP-4Cl:PBDB-TF (BHJ). **c** Absorption spectra of Cr/Ce/Mn-LC and BHJ and CsPbI_3_:Er^3+^ PQDs and CsPbI_3_:Er^3+^ PQDs/BHJ film, and photoluminescence spectra of Cr/Ce/Mn-LC

Figure 1a and S1 illustrates the structure and the cross-sectional scanning electron microscopy (SEM) images of the broadband PDs, which consists of CsPbCl_3_:Cr^3+^,Ce^3+^,Mn^2+^ PQDs doped polymethyl methacrylate LC (Cr/Ce/Mn-LC)/ITO/SnO_2_:Ti_3_C_2_/CsPbI_3_:Er^3+^ PQDs/PBDB-TF:BTP-4Cl (BHJ)/Ag. Firstly, the SnO_2_:Ti_3_C_2_ ETL (~50 nm) was spin-coated on ITO substrate and then annealed at 150 °C for 15 min. The CsPbI_3_:Er^3+^ PQDs with a thickness of 450 nm were fabricated on the ETL modified ITO glass by spin-coating. Then the organic BHJ of PBDB-TF: BTP-4Cl as an NIR photosensitive layer was deposited on top of the CsPbI_3_:Er^3+^ PQDs film. Finally, the Cr/Ce/Mn-LC was positioned at the ITO side to construct the UV-Visible-NIR PD. The mixture of BHJ can absorb the low-energy NIR photons and effectively passivate the defects in the perovskite film and improve carrier transport and collection (Fig. 1b)^43^. As demonstrated in Fig. 1c, CsPbI_3_:Er^3+^ PQDs has good absorption in the range of 350–700 nm, BHJ exhibits light absorption in the range of 700–1000 nm, and the CsPbI_3_:Er^3+^ PQDs/BHJ film shows the absorption band from 350 to 1000 nm. Differently, the detected lights with a wavelength within 200–400 nm, are completely absorbed by Cr/Ce/Mn-LC and converted to 400–700 nm, which are further absorbed by CsPbI_3_:Er^3+^ PQDs. Finally, combining UV-Visible-NIR absorption of Cr/Ce/Mn-LC and CsPbI_3_:Er^3+^ PQDs and BHJ, the fabricated PDs can exhibit a wide photodetection range from 200 to 1000 nm.

**Fig. 2:**
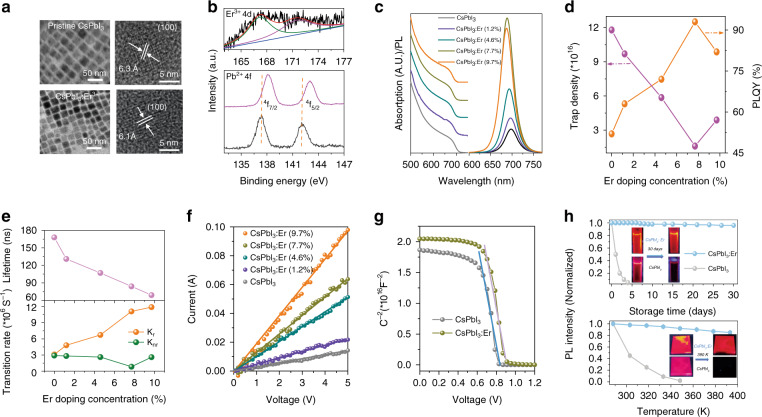
Er^3+^ doped CsPbI_3_ PQDs. **a** TEM and HR-TEM images of CsPbI_3_ and CsPbI_3_: Er^3+^ (7.7%) PQDs. **b** XPS spectra of Er^3+^ (4d) and Pb^2+^ (4f) of CsPbI_3_ and CsPbI_3_: Er^3+^ (7.7%) PQDs. Absorption and emission spectra (**c**), trap density and PLQY (**d**), and PL decay lifetimes and transition rates (**e**) of CsPbI_3_ and CsPbI_3_:Er^3+^ PQDs with different Er^3+^ concentrations of 1.2%, 4.6%, 7.7%, 9.7%. **f** *I*–V curves of CsPbI_3_ and CsPbI_3_:Er^3+^ PQDs as a function of different Er^3+^ concentrations in ITO/ETL/ PQDs /Ag devices. **g** Mott–Schottky measurement of CsPbI_3_ and CsPbI_3_: Er^3+^ (7.7%) PQDs. **h** Normalized PL intensity of CsPbI_3_ and CsPbI_3_: Er^3+^ (7.7%) PQDs as a function of storage time and temperature; Inset is PL images of CsPbI_3_ and CsPbI_3_: Er^3+^ (7.7%) PQDs after 30 days and 390 K under 365 nm UV lamp

Firstly, the visible sensitive materials of CsPbI_3_ and CsPbI_3_:Er^3+^ PQDs with different Er^3+^ doping concentrations (0–9.7%) were synthesized through the modified hot-injection method^31,44^. As revealed in a transmission electron microscope (TEM) and high-resolution TEM (HRTEM) images (Fig. 2a and S2), the cubic-shaped and uniform CsPbI_3_ PQDs are successfully obtained with and without Er^3+^ doping. The average diameter of pristine CsPbI_3_ PQDs is determined to be 11.4 nm, which gradually decreases to 10.9, 10.7, 10.3, and 9.9 nm with different Er^3+^ doping concentrations (1.2, 4.6, 7.7, and 9.7%), respectively (Fig. S3). The doping amounts of Er^3+^ in the CsPbI_3_ PQDs were ascertained by the inductively coupled plasma optical emission spectrometry (ICP-OES) (Table S1). Meanwhile, the lattice constant of the (100) plane of the CsPbI_3_:Er^3+^ (7.7%) PQDs is (6.1 Å), smaller than that of CsPbI_3_ PQDs (6.3 Å). This can be attributed to the lattice contraction of PQDs induced by partly replacing the Pb^2+^ (~119 pm) with the doping Er^3+^ ions (~88.1 pm). The X-ray diffraction (XRD) patterns evidence that CsPbI_3_:Er^3+^ PQDs has the same cubic structure as CsPbI_3_ PQDs, and the diffraction peaks of (100) and (200) planes shift toward higher diffraction angle after Er^3+^ doping (Fig. S4). The energy-dispersive X-ray (EDX) mapping images reveal that all the elements (Cs, Pb, I, and Er) exist in CsPbI_3_:Er^3+^ (7.7%) PQDs (Fig. S5). The X-ray photoelectron spectra (XPS) of pristine CsPbI_3_ and CsPbI_3_:Er^3+^ PQDs in Fig. 2b and S6 demonstrate that the peaks of Cs 3*d*, Pb 4*f*, and I 3*d* are identified in both two samples, and two additional peaks are observed appearing at 167.6 and 172.1 eV in CsPbI_3_:Er^3+^ PQDs, which can be assigned to the 4*d* signal of Er^3+^. Compared with CsPbI_3_ PQDs, the binding energy of Pb^2+ 4^*f*_5/2_ and ^4^*f*_7/2_ in CsPbI_3_:Er^3+^ PQDs shifts to higher energy, while remains unchanged for I^−^ 3*d* and Cs^+^ 3*d*. When doping with Er^3+^, the lattice contraction of CsPbI_3_ PQDs happens induced by partly replacing the Pb^2+^ (~119 pm) with the smaller sized Er^3+^ (~88.1 pm), leading to the shrinking of PbI_6_ octahedron with shortening of Pb-I bond and enhancing the interaction^45,46^. Thus, the binding energy of Pb^2+^ 4*f* in PQDs shifts with Er^3+^ doping, while remains unchanged for I^−^ 3*d* and Cs^+^ 3*d*. Combined with the HRTEM (Fig. 2a) and XRD results (Fig. S5), we can deduce that Er^3+^ ions are successfully incorporated into the CsPbI_3_ PQDs and occupy the lattice position of Pb^2+^ ions. Similar results were reported in the previous literatures^19,47,48^.

Figure 2c shows the absorption and emission spectra of CsPbI_3_ and CsPbI_3_:Er^3+^ PQDs. The absorption peak at 681 nm can be observed, attributed to the intrinsic exciton transition of CsPbI_3_ PQDs. With increasing Er^3+^ doping concentration, the absorption peak of CsPbI_3_ PQDs gradually shifts from 681 to 672 nm, and the bandgap of PQDs increases from 1.73 eV (CsPbI_3_ PQDs) to 1.81 eV (CsPbI_3_: 9.7% Er^3+^ PQDs), as represented in Fig. S7. This can be mainly attributed to the lattice contraction by substituting Pb^2+^ ions with Er^3+^ ions (Supplementary Note 1), as observed in Fig. 2a. Similarly, the emission peak of CsPbI_3_ PQDs gradually blue shifts from 697 to 687 nm with increasing Er^3+^ doping. Interestingly, the PLQY of PQDs rapidly increases after Er^3+^ doping, from 52% for CsPbI_3_ PQDs to 93% for CsPbI_3_: Er^3+^ (7.7%) PQDs (Fig. 2d). Correspondingly, the trap density of CsPbI_3_ PQDs is estimated to be 1.18 × 10^17^ cm^−3^, which quickly decreases to 1.6 × 10^16^ cm^−3^ for CsPbI_3_: Er^3+^ (7.7%) PQDs (Fig. S8 and Supplementary Note 2). Meanwhile, the carrier densities are evaluated as 1.404 × 10^19^ cm^−3^ for CsPbI_3_ PQDs and 1.621 × 10^19^ cm^−3^ for CsPbI_3_: Er^3+^ (7.7%) PQDs. It is found that the effect reduces the trap density and increases the carrier densities of PQDs after Er^3+^ doping, similar to the previous literatures^49,50^.

The Er^3+^ doping concentration dependence of PL lifetimes (τ) and transition rates of CsPbI_3_ and CsPbI_3_:Er^3+^ PQDs were measured (Fig. 2e and S9). It can be observed that the PL lifetimes gradually decrease from 168 ns of CsPbI_3_ PQDs to 69 ns of CsPbI_3_:Er^3+^ (9.7%) PQDs. The radiative rates (*k*_r_) and nonradiative rates (*k*_nr_) of CsPbI_3_ and CsPbI_3_:Er^3+^ PQDs were calculated in Table S2 according to the following equations: *k*_r_ = PLQY/τ, and *k*_nr_ = (1-PLQY)/τ. Compared to the CsPbI_3_ PQDs, the k_r_ of CsPbI_3_:Er^3+^ (7.7%) PQDs increases about 3.6-folds and the k_nr_ of CsPbI_3_:Er^3+^ (7.7%) PQDs decreases about 3.5-folds. It suggests that the Er^3+^ doping can boost the radiative decay rate, reduce the trap density and enhance the PLQY of PQDs. The increase of the radiative decay rate in CsPbI_3_:Er^3+^ (7.7%) PQDs can be proved by the decreased power index as a function of the excitation power density (Fig. S10)^51^.

The role of Er^3+^ in the electrical conductivity of PQDs films were studied using *I–V* curves of the ITO/PQDs /Ag devices (Fig. 2f and Supplementary Note 3). It can be seen that the conductivity (*σ*) of pristine CsPbI_3_ PQDs is 1.2 × 10^–6^ S cm^−1^, which increases to 5.2 × 10^−6^ S cm^−1^ for CsPbI_3_:Er^3+^ (7.7%) PQDs. The built-in potential (*V*_*b*_) values of CsPbI_3_ and CsPbI_3_:Er^3+^ (7.7%) PQDs based devices are estimated to be 0.85 and 0.93 V by the Mott–Schottky analysis (Fig. 2g and Supplementary Note 4). A larger built-in potential value means an enhanced driving force for the separation of photogenerated carriers as well as an extended depletion region for efficient suppression of electron-hole recombination, which is favorable for carrier separation, transport, and extraction^52–54^. The built-in potential (*V*_*b*_) values of CsPbI_3_:Er^3+^ PQDs is larger than that of CsPbI_3_ PQDs, realizing the more effective separation of photogenerated carriers after Er^3+^ doping.

It should be highlighted that incorporating Er^3+^ ions into CsPbI_3_ PQDs demonstrates significantly enhanced air- and UV- stability, which is of great importance for practical applications^35,55,56^. The PL intensity of CsPbI_3_:Er^3+^ (7.7%) PQDs still maintains above 97 and 67% of its initial value after 30 days of storage and 10 h UV light radiation, but the PL of CsPbI_3_ PQDs almost disappears after 5 days storage and 8 h UV light radiation (Fig. 2h and Figs. S11, S12). The thermal stability of CsPbI_3_ PQDs is also improved remarkably after Er^3+^ doping, which PL intensity remains 87% after annealing at 390 K, and no PL is recorded for CsPbI_3_ PQDs after annealing at 350 K. Considering the better conductivity, the CsPbI_3_ and CsPbI_3_:Er^3+^ (7.7%) PQDs are further treated with ethyl acetate to remove the original long-chain ligands of oleic acid (OA) and oleylamine (OAm) of PQDs^57,58^. The CsPbI_3_:Er^3+^ PQDs demonstrate outstanding air- and UV- stability (Fig. S13).

**Fig. 3:**
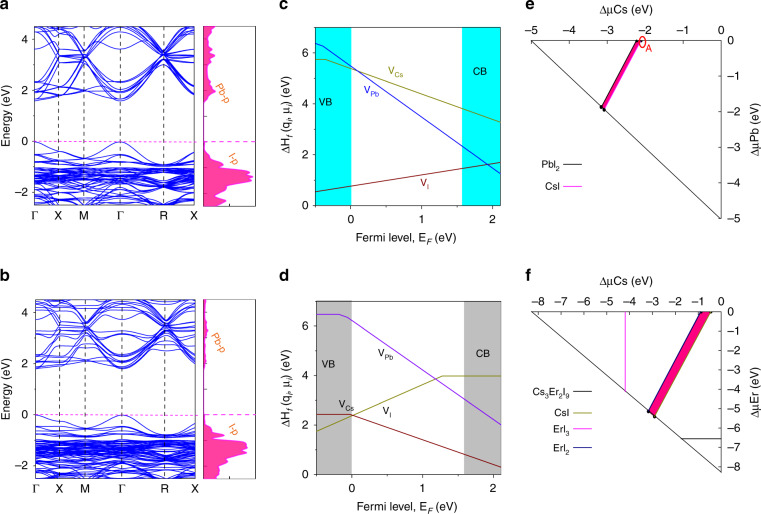
Density functional theory calculations of CsPbI_3_ and CsPbI_3_:Er^3+^ PQDs. **a**, **b** Band structure and DOS of CsPbI_3_ and CsPbI_3_:Er^3+^ PQDs. **c**, **d** Calculated formation energy of intrinsic vacancy defects for Cs, Pb and I (labeled as VCs, VPb and VI) of CsPbI_3_ and CsPbI_3_:Er^3+^ PQDs. **e**, **f** Calculated phase diagrams of cubic CsPbI_3_ (8 fu/cell) and CsErI_3_ (8 fu/cell) with internal symmetry breaking. The pink polygons indicate the stable regions of the single-phase of cubic CsPbI_3_ and CsErI_3_

We next performed density functional theory (DFT) calculations aimed at understanding the origin of Er^3+^ doping-induced changes in structural and photophysical properties of CsPbI_3_ PQDs. Electronic structures and formation energy of intrinsic vacancies with different charge states (q_i_) at Pb rich and I poor condition in pure cubic CsPbI_3_ and Er^3+^ doped CsPbI_3_ were calculated using Perdew–Burke–Ernzerhof (PBEsol) functional without considering the spin-orbit coupling effect (Supplementary Note 5)^59^. Fig. 3a illustrate the electronic structure and density of states (DOS) of pure cubic CsPbI_3_ (2 × 2 × 2 supercell, 8fu/cell) with direct bandgap 1.72 eV and internal symmetry breaking. The I 5*p* and Pb 6 *s* orbital mainly contribute to the valance band maximum (VBM), and Pb 6*p* and I 5*p* dominate the conduction bands minimum (CBM). As shown in Fig. 3b, the bandgap of CsPbI_3_:Er^3+^ is larger than pristine CsPbI_3_, and the band edge states of CsPbI_3_:Er^3+^ (8 fu/cell) does change (i.e., VBM and CBM), presenting the bandgap increase to 1.82 eV. We also theoretically calculated the formation energy of intrinsic vacancy defects of Cs, Pb, and I (labeled as V_Cs_, V_Pb_, and V_I_) in pure cubic CsPbI_3_ and CsPbI_3_:Er^3+^ (64 fu/cell) by using PBEsol functional and 2 × 2 × 2 k-grid. As illustrated in Fig. 3c, d and Tables S3, S4, the intrinsic vacancies in CsPbI_3_ are shallow defects with relatively smaller formation energy, which shows larger formation energy in CsPbI_3_:Er^3+^, leading to the reduced trap density after Er^3+^ doping, similar to the experimental results in Fig. 2d. Generally, the defects can act as carrier traps, resulting in nonradiative recombination, whereas fewer defects largely preserve the bulk electronic band structure and can improve the optoelectronic properties of PQDs^31,60^. Table 1 lists the formation energy of ternary compounds to the corresponding binary compounds by DFT. The calculated formation energy ΔH_f_ of CsPbI_3_:Er^3+^ reveals that the reaction spontaneously occurs starting from binary precursors because of the exothermic reaction. The reaction path is: CsI + Er^3+^I_2_ + e + PbI_2_ -> Cs_8_Pb_7_ErI_24_ + e. The calculated formation energy ΔH_f_ (defined in Table 1) of Er^3+^ doped CsPbI_3_ with respect to binary precursors CsI, Er^3+^I_2_, and PbI_2_ is positive, referring to the exothermic reaction^61^. This indicates that the reaction CsI + 0.077(ErI_3_) + 0.923(PbI_2_) - >CsPb_0.923_Er_0.077_I_3_ spontaneously can occur. The formation energy of CsPbI_3_:Er^3+^ PQDs is larger than prsitine CsPbI_3_ PQDs, which demonstrate the CsPbI_3_:Er^3+^ PQDs are more energetically stable than CsPbI_3_ PQDs. Moreover, the chemical potential in 2 × 2 × 2 supercell of cubic CsPbI_3_ was calculated by considering the binary competing phase CsI and PbI_2_. As illustrated in Fig. 3e, f, the chemically stable range for cubic CsPbI_3_ is smaller than CsErI_3_. Meanwhile, the formation energy and tolerance factors of CsPbI_3_ become larger after Er^3+^ doping (Table S1 and Fig. S14), implying the more energetically stable structure of CsPbI_3_:Er^3+^ (Fig. 2h). Experimentally, the reduced defect density, the enhanced carrier densities and the conductivity, and the increased built-in potential (*V*_*b*_) values of CsPbI_3_ PQDs after Er^3+^ doping were observed. Therefore, experimental and theoretical results demonstrate that the Er^3+^ doping can reduce trap density, improve the density and mobility of carriers, accelerate carriers' separation, and enhance the stability of CsPbI_3_ PQDs.

**Table 1:** Formation energy of ternary compounds (CsPbI_3_, and Er^3+^ doped CsPbI_3_) to binary compounds calculated by using PBEsol functional

Compound	Phase	${{\Delta }}H_f = E_{BX_2} + E_{AX} - E_{ABX_3}$(eV/atom)
CsPbI_3_	Cubic (8fu/cell)	0.015
CsPb_0.923_Er_0.077_I_3_	Cubic (8fu/cell)	0.019
CsErI_3_	Cubic (8fu/cell)	0.047
Cs_3_Er_2_I_9_	Hexagonal (2fu/cell)	0.050

**Fig. 4:**
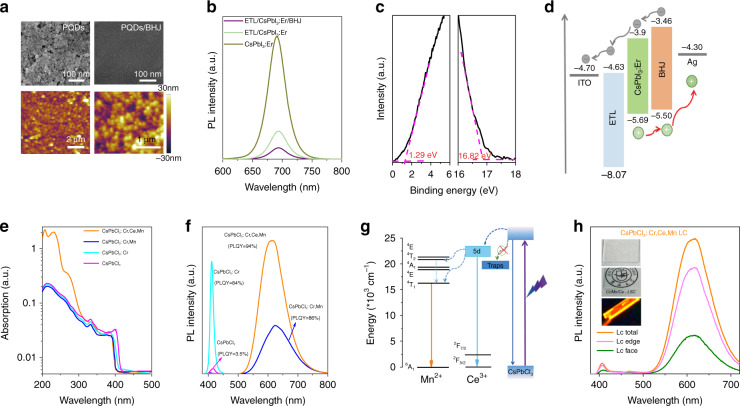
Photoelectric characteristics of BHJ and Cr/Ce/Mn-LC. **a** SEM (top) and AFM (bottom) images of the CsPbI_3_:Er^3+^ (7.7%) PQDs film and the CsPbI_3_:Er^3+^ (7.7%) PQDs /BHJ film. **b** Emission spectra of CsPbI_3_:Er^3+^ (7.7%) PQDs film, ETL/CsPbI_3_:Er^3+^ (7.7%) PQDs film, and ETL/CsPbI_3_:Er^3+^ (7.7%) PQDs/BHJ film. **c** UPS curves of CsPbI_3_:Er^3+^ (7.7%) PQDs film. **d** Energy band diagram of each layer in the PDs. **e**, **f** Absorption spectra, emission spectra, and PLQY of undoped, Cr^3+^, Cr^3+^/Mn^2+^, and Cr^3+^/Mn^2+^/Ce^3+^ doped CsPbCl_3_ PQDs. **g** Energy transfer mechanism of CsPbCl_3_: Cr^3+^, Ce^3+^, Mn^2+^ PQDs. **h** Total, face, and edge emissions measured for a Cr/Ce/Mn-LC using Cr^3+^,Mn^2+^,Ce^3+^ doped CsPbCl_3_ PQDs. The inset is the picture of Cr/Ce/Mn-LC under sunlight and UV illumination

Figures 4a and S15 display the SEM and atomic force microscopy (AFM) images of CsPbI_3_:Er^3+^ (7.7%) PQDs film and the CsPbI_3_:Er^3+^ (7.7%) PQDs/BHJ film. It is found that the CsPbI_3_:Er^3+^ PQDs / BHJ film exhibited smaller roughness with root mean square 6.75 than 8.5 nm for the CsPbI_3_:Er^3+^ (7.7%) PQDs film. When combining CsPbI_3_:Er^3+^ (7.7%) PQDs with BHJ to form heterogeneous conjunctiva, the hybrid film becomes more smooth, which provides a high-speed transport channel for carriers and promotes the dissociation of excitons^58,62–64^. As expected in Fig. 4b, the CsPbI_3_:Er^3+^ (7.7%) PQDs film shows a strong red emission band located at 685 nm. The PL intensity of CsPbI_3_:Er^3+^ (7.7%) PQDs significantly decreases after integrated ETL and BHJ, attributed to the enhanced charge extraction and suppressed carrier recombination in CsPbI_3_:Er^3+^ (7.7%) PQDs film. The ultraviolet photoemission spectroscopy (UPS) was performed to confirm the energy level locations for CsPbI_3_:Er^3+^ (7.7%) PQDs (Fig. 4c), in which the Fermi level shifts to −4.37 eV, and the CBM and VBM values were determined to be −3.90 and −5.69 eV after Er^3+^ doping. As revealed in Fig. S16a, b, the alignment of the highest occupied molecular orbital (HOMO) levels and the lowest unoccupied molecular orbital (LUMO) of BHJ were −5.50 and −3.46 eV, which presents the secondary electron cutoffs and valence band edges of the PBDB-TF (donor) and BTP-4Cl (acceptor), respectively. Figures 4d and S16c illustrate the energy diagram of the PD device with the structure of ITO/ETL/CsPbI_3_:Er^3+^ (7.7%) PQDs/BHJ/Ag. The CsPbI_3_:Er^3+^ (7.7%) PQDs film absorbs visible photons, and the generating carriers separate at the interface of ETL/CsPbI_3_:Er^3+^ (7.7%) PQDs film and CsPbI_3_:Er^3+^ (7.7%) PQDs/BHJ film. Meanwhile, the BHJ captures the low-energy photons (e.g., NIR light) to produce electrons and holes, and the electrons transported to ETL through CsPbI_3_:Er^3+^ (7.7%) PQDs film, and the holes move to Ag electrode.

To obtain Cr/Ce/Mn-LC, the homogeneous and cubic Cr^3+^, Cr^3+^/Mn^2+^, and Cr^3+^/Mn^2+^/Ce^3+^ doped CsPbCl_3_ PQDs were successfully prepared by the modified hot-injection method^37,65^ as revealed in TEM images, XRD patterns, and XPS spectra in Figs. S17–19. Figure 4e shows the UV-Vis absorption spectra of pristine and doped CsPbCl_3_ PQDs. It presents that the absorption peak of CsPbCl_3_ PQDs locates at 400 nm ascribed to the excitonic transition, and slightly shifts to a shorter wavelength after Cr^3+^,Mn^2+^,Ce^3+^ doping into CsPbCl_3_ PQDs. Interestingly, the absorption of Cr^3+^,Mn^2+^,Ce^3+^ doped CsPbCl_3_ PQDs increases remarkably at the range of 200–300 nm, which can be assigned to the high states of 5*d* of Ce^3+^, similarly to the previous literature^37,66^. As displayed in Fig. 4f, one emission band (417 nm) in CsPbCl_3_ and CsPbCl_3_:Cr^3+^ PQDs is observed, while two emission peaks in CsPbCl_3_:Cr^3+^, Mn^2+^, and CsPbCl_3_:Cr^3+^, Mn^2+^, Ce^3+^ PQDs are identified, appearing at 415 and 590 nm, respectively, corresponding to exciton emission of PQDs and ^4^T_1_ - ^6^A_1_ transition of Mn^2+^^67^. The Mn^2+^ emission originates form the energy transfer from PQDs to Mn^2+^ ions, evidenced by the decreased decay lifetimes of excitation emission in CsPbCl_3_:Cr^3+^ PQDs after Mn^2+^ doping (Fig. S20). Surprisingly, the PLQY of CsPbCl_3_:Cr^3+^, Mn^2+^, Ce^3+^ PQDs is measured to be 93.5%. According to the previous literatures^68,69^, such high PLQY (Fig. 4f) are mainly due to the reduced nonradiative decay rate after Cr^3+^ doping, the boosted energy transfer from PQDs to Mn^2+^ ions and the enhanced UV absorption after Ce^3+^ doping. The energy transfer mechanism of CsPbCl_3_:Cr^3+^, Mn^2+^, Ce^3+^ PQDs is presented in Fig. 4g. Importantly, the doping with Cr^3+^,Mn^2+^,Ce^3+^ can also largely improves the stability of CsPbCl_3_ PQDs, in which the PL intensity remains around 93% after 30 days (Fig. S21). In virtue of high conversion efficiency from UV to visible lights, the CsPbCl_3_:Cr^3+^, Mn^2+^, Ce^3+^ PQDs are embedded into a PMMA polymer matrix to form a Cr/Ce/Mn-LC (inset of Fig. 4h). The Cr/Ce/Mn-LC demonstrates the similar emission spectra with CsPbCl_3_:Cr^3+^,Mn^2+^,Ce^3+^ PQDs (Fig. 4h and S22) and high transparency to visible lights (Fig. S23). The face emission of the PLQY Cr/Ce/Mn-LC is 20.03%, which is lower than the edge emissions (61.47%) due to the total internal reflection to the edges in the LC (Fig. S24). Such Cr/Ce/Mn-LC processes the high efficiency and transparency, can be severed as a photoluminescent converter to boost the UV response of PD.

**Fig. 5:**
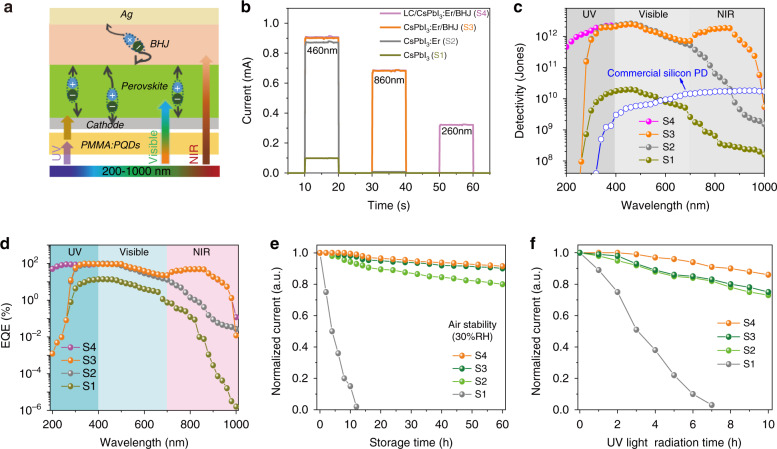
Performance of broadband PDs. **a** Charge generation and transport mechanism of a broadband PD. **b** Photocurrents of the ITO/ETL/CsPbI_3_/Ag (S1), ITO/ETL/CsPbI_3_:Er^3+^ (7.7%) PQDs/Ag (S2), ITO/ETL/CsPbI_3_:Er^3+^ (7.7%) PQDs/BHJ/Ag (S3) and Cr/Ce/Mn-LC/ITO/ETL/CsPbI_3_:Er^3+^ (7.7%) PQDs/BHJ/Ag (S4) devices under the 260 nm, 460 nm, and 860 nm, respectively. **c** D* of the S1–S4 devices and commercial Silicon PD. **d** EQE of the S1–S4 devices. **e**, **f** Stability of the S1–S4 devices under 30% RH and UV light radiation

Figure 5a shows the charge generation and transport mechanism of the broadband PDs with the response range spanning from UV to NIR lights, in which PQDs straightly absorb the visible lights, the BHJ layer captures the NIR lights, Cr/Ce/Mn-LC converts the UV lights (200–400 nm) to visible lights (400–700 nm) further absorbing by PQDs. The photocurrent– time (I–t) response curves based on the ITO/ETL/CsPbI_3_ PQDs/Ag (S1), ITO/ETL/CsPbI_3_:Er^3+^ (7.7%) PQDs/Ag (S2), ITO/ETL/CsPbI_3_:Er^3+^ (7.7%) PQDs/BHJ/Ag (S3) and Cr/Ce/Mn-LC/ITO/ETL/CsPbI_3_:Er^3+^ (7.7%) PQDs/BHJ/Ag (S4) under the 260, 460, and 860 nm with an incident light intensity of 0.5 mW cm^−2^ are recorded in Fig. 5b. The photocurrents reach 0.89, 0.68, and 0.32 mA of S4 PD, while they are only 0.098 mA, 1.4 µA, and 0.01 µA of S1 PD under 460, 860, and 260 nm, respectively. Compared with S1 PD, the photocurrents of S4 PD enhance ninefold for 460 nm, 485-folds for 860 nm, and 3.2 × 10^4^ folds for 260 nm. The improvement of photocurrent at 460 nm mainly originates from that the Er^3+^ doping into CsPbI_3_ PQDs resulting in increased carriers mobility, and accelerated carriers transport (Fig. 2). BHJ hybridization not only significantly boosts the photocurrent of 860 nm owing to the direct NIR absorption, but also contributes to the slight increase of photocurrent at 460 nm (S3) due to the inhibited electron-hole pair recombination at the interfaces of CsPbI_3_:Er^3+^ (7.7%) PQDs/BHJ film. The huge enhancement of photocurrent at 260 nm (S4) is on account of Cr/Ce/Mn-LC. In order to further demonstrate highly light emission efficiency of the Cr/Ce/Mn-LC on the side edge, the photocurrent–time (I–t) response curves based on the Cr/Ce/Mn-LC face and the edge of S4 PDs at 260 nm illumination with an incident light intensity of 0.5 mW cm^−2^ are recorded in Figs. S25, 26. It can be seen that the photocurrent of Cr/Ce/Mn-LC surface is 0.092 mA, which increases to 0.32 mA for the side edge of S4 PDs. The improvement of photocurrent at 260 nm mainly originates from the waveguide structure and the highly improved light collecting efficiency^70^. Moreover, the photocurrent response of PDs using PMMA: CsPbCl_3_: Cr^3+^, Mn^2+^, Ce^3+^ luminescent conversion layer (0.105 mA) is also lower than that of Cr/Ce/Mn-LC (0.32 mA). In the luminescent conversion layer, the direction of visible emissions are random under UV illumination, and only the emission photons which irradiate on the PD can be utilized. But in the Cr/Ce/Mn-LC, most of the emission photons can be traveled to the edge side attached to PD. Thus, the improvement of photocurrent in Cr/Ce/Mn-LC mainly originates from the waveguide structure and the highly improved light (400–700 nm) concentrate efficiency. The dark currents of the S2–S4 PDs are lower than that of the pristine PD device by one to two orders of magnitude, suggesting the defect sites of PQDs are effectively passivized by Er^3+^ doping and BHJ hybridization (Fig. S27), consistently with the results in Figs. 2, 4a–d^46,71^. In line with the decreased dark current of S3 and S4 PDs, the *θ* coefficients in the S3 and S4 PDs (*θ* = 0.763 and 0.767) have better linearity than the pristine PD (*θ* = 0.698), following the power-law *I* ∼ *P*^θ^, where *I* and *P* represent the photocurrent and the incident light power intensity (Fig. S28)^72^. Figs. 5c, d and S29 display the detectivity (D*), external quantum efficiency (EQE), and photoresponsivity (R) of S1–S4 PDs. These three parameters satisfy the following equations^20,37^:

1 $${{{\mathrm{R}}}} = \frac{{I_{ph} - I_d}}{{PS}}$$

2 $${{{\mathrm{D}}}} \ast = \sqrt {\frac{S}{{2eI_d}}} {{{\mathrm{R}}}}$$

3 $${{{\mathrm{EQE}}}} = {{{\mathrm{R}}}}\frac{{hc}}{{\lambda e}}$$

where *I*_*ph*_ and *I*_*d*_ are the photocurrents under the illumination of light and in the dark, *P* and *S* are the input light power density and the effective irradiated area, *h* and *c* are the Planck’s constant and the speed of light, λ and *e* are the incident light wavelength and the elementary charge. The pristine S1 PD presents the low D*, EQE, and R in the whole region. Those values are largely boosted in the visible region in S2–S4 PDs, and simultaneously, its responses expand to NIR (S3 PD) and UV (S3 and S4 PD) regions. The R, EQE, and D* of S4 PDs are 266.2, 439.8, and 326 mA/W; 89.13, 91.84, and 47.12%; 1.14 × 10^12^ Jones, 2.46 × 10^12^ Jones, 1.82 × 10^12^ Jones at 260, 460, and 860 nm light detection, respectively. Compared with S1 PD, owing to the contribution of Er^3+^ doping, the BHJ film, and Cr/Ce/Mn-LC, the D* of S4 PDs improves 1.2 × 10^4^ folds, 1.24 × 10^2^ folds, and 5.7 × 10^3^ folds at 260, 460, and 860 nm lights, which increases two to four orders than that of the pristine PDs. Moreover, the S4 PDs exhibits a fast response time (R_t_) of hundreds of microseconds (Fig. S30). The long-term stability of the S1–S4 PDs was further studied. As displayed in Fig. 5e, the S2–S4 PDs maintain about 82% of the initial photocurrent, while the photocurrent of the S1 device dropped to 0% after 60 h, owing to the outstanding stability of PQDs by Er^3+^ doping. The UV stability of the S1–S4 devices in Fig. 5f illustrates that the stability of S2–S4 PDs are large improved, especially, S4 PD represents the best UV light stability, maintaining above 86% of the initial photocurrent after 10 h UV illumination, but it degrades to 0% for pristine PD within 7 h. The reason for those improvements in the air- and UV- stability are mainly attributed to the role of Er^3+^ doping, BHJ hybridization, and the buffer layer of Cr/Ce/Mn-LC in the device. Notably, the photocurrent of the PD is repeatable even after five thousand cycles (Fig. S31), confirming the excellent reversibility of this photodetector. Compared to the previous broadband perovskite PDs (Table 2), our device exhibits excellent performance with a relatively wide response, high responsivity, and detectivity, especially in UV and NIR regions, and good stability, which exceeds the results of the previous reports.

**Table 2:** Figures-of-merit in typical perovskite PDs

Materials	D* (Jones)	*R*_*t*_ (μs)	Wavelength	EQE	Ref.
CH_3_NH_3_PbI_3-x_Cl_x_	−	6.5/5.0	400–800	80%	2
TiO_2_ nanorods/MAPbI_3_ heterojunction	7.8 × 10^10^	10^6^	300–800	−	11
MAPbI_3_	10^11^	2200/4000	265–800	−	55
PEIE/CsPbIBr_2_	9.7 × 10^12^	−	400–600	57.1%	12
OTPD/MAPbI_3_	7.4 × 10^12^ @680 nm	−	300–780	80%	5
NiO_x_/MAPbI_3_/PCBM /PTB7-Th:IEICO-4F	~10^10^	500/510	340–940	80%/70%	35
P3HT/PDPP3T/ CsPbBr_3_/SnO_2_	1.2 × 10^13^	92/193	300–950	−	7
PEDOT:PSS/PbS/CsPbCl_3_	5.78 × 10^10^	−	350–1100	−	32
Au/PbS/CsPbCl_3_	3.29 × 10^13^	2.7 × 10^5^/4.2 × 10^5^	300–1100	10^6^%	33
Cr/Ce/Mn-LC/ITO /ETL/ Er^3+^ doped CsPbI_3_ PQDs /BHJ/Ag	2.46 × 10^12^ @ 460 nm 1.85 × 10^12^ @870 nm 1.14 × 10^12^ @ 260 nm	350/300	200–1000	91.8%	Our work

## Discussion

In this work, unique broadband PDs with the response range of 200–1000 nm and the D* value reaching of 1.14 × 10^12^ at 260 nm and 2.46 × 10^12^ at 460 nm, and 1.85 × 10^12^ at 860 nm based on doped PQDs and an organic bulk heterojunction and Cr/Ce/Mn-LC were reported. Several new contributions for developing broadband PDs in this work should be highlighted. Firstly, CsPbI_3_:Er^3+^ PQDs serve as a visible photosensitive layer of PD, and the performance improves two orders that of pristine PDs at the wavelength of 400–700 nm. Experimental and theoretical results demonstrate that the highly improved optoelectronic properties, such as low trap density, high charge mobility, and PLQY, excellent stability through Er^3+^ doping, lead to the great performance of PD. Secondly, the hybridization of CsPbI_3_:Er^3+^ PQDs / BHJ successfully expands the response of PDs to the NIR region, due to the NIR light-absorbing of low bandgap organic BHJ film. Thirdly, the high efficiency (PQLY = 93.5%) and strong absorption ability of Cr^3+^, Mn^2+^, Ce^3+^ doped CsPbCl_3_ PQDs are incorporated into PMMA to form a Cr/Ce/Mn-LC. The Cr/Ce/Mn-LC is employed as a UV photosensitive layer of PDs to highly enhanced UV response of PDs. Furthermore, the air- and UV- stability of broadband PDs are also significantly boosted. The performance of our PD is much better than that of other perovskite PDs and commercial Si PDs. Overall, this work presents a novel strategy to obtain a broadband PD with excellent performance.

## Materials and methods

### Materials

Cs_2_CO_3_ (99.9%), 1-octadecene (ODE, 90%), oleic acid (OA, 85%), oleylamine (OAm, 70%), PbI_2_ (99.99%), ErI_3_ (99.99%), PbCl_2_ (99.9%), CrCl_3_ (99%), MnCl_2_ (99%), toluene, and ethyl acetate (99%) were purchased from Sigma-Aldrich and were used as starting materials without further purification.

### Synthesis of Cs-oleate

About 0.8 g Cs_2_CO_3_ was added into a mixture of 30 mL of ODE and OA (2.5 mL) and then heated to 150 °C and the white powder was completely dissolved. The mixture was then kept at 120 °C.

### Synthesis of CsPbI_3_ PQDs

PbI_2_ (0.3 mmol), OAm (1.5 mL), OA (1.5 mL), and ODE (10 mL) were added to a 50-mL three-neck round-bottomed flask and were evacuated and refilled with N_2_, followed by heating the solution to 120 °C for 1 h. The temperature of the solution was then increased to 180 °C for 10 min. Then, the Cs-oleate (1 mL) was swiftly injected into the solution. After 10 s, the solution was cooled in an ice bath. The CsPbI_3_ PQDs were precipitated and then centrifuged, followed by dissolution in toluene.

### Synthesis of Er^3+^ doped CsPbI_3_ PQDs

PbI_2_ (0.3 mmol) and ErI_3_ (0.15 mmol) were loaded into round-bottom flask with OAm (1.5 mL), OA (1.5 mL), and ODE (10 mL). It was continued heated at 120 °C for 2 h and refilled with N2. Then the solution was increased to 230 °C. Then, the Cs-oleate (1 mL) was swiftly injected into the solution. After 10 s, the solution was cooled in an ice bath. Finally, the CsPbI_3_:Er^3+^ (7.7 %) PQDs were precipitated and then centrifuged, followed by dissolution in toluene.

### Synthesis of Cr^3+^,Ce^3+^,Mn^2+^ doped CsPbCl_3_ PQDs

PbCl_2_ (0.5 mmol), CrCl_3_ (0.3 mmol), CeCl_3_(0.2 mmol), and MnCl_2_ (0.2 mmol) were loaded into round-bottom flask with OAm (1.5 mL), OA (1.5 mL), and ODE (15 mL). The following steps were the same with the synthesis of the Er^3+^ doped CsPbI_3_ PQDs.

### Fabrication of Cr/ Ce /Mn –LC

About 0.8 g PMMA (MW ~350000) was dispersed in 5 mL toluene by sonication, to which 2.5 mL toluene solution of CsPbCl_3_: Cr^3+^(8.3%),Ce^3+^(3.2%),Mn^2+^(9.3%) PQDs were added. The mixture was sealed and stirred overnight to obtain a homogenous slurry. The slurry was centrifuged at 2000 rpm and the supernatants were used for LC fabrication. The above supernatants were dropped onto borosilicate glass substrates and LC was fabricated by spin-coating.

### Syntheses of BHJ film

The PBDB-TF:BTP-4Cl (1:1.2) was dissolved in chloroform. The mixture was heated and stirred at 60 °C for 5 h to obtain an organic active layer solution. The solvent additive of 1-chloronaphthalene (CN) (0.5%) was added half an hour before the organic active layer solution deposition. For the hybrid PDs, the PBDB-TF:BTP-4Cl solution was spin-coated on a perovskite layer at 1800 rpm for 60 s and subsequently annealed at 80 °C for 10 min.

### Device fabrication

ITO-coated glass substrates were etched with zinc powder and HCl to define the electrode patterns and washed in deionized water, acetone, and ethanol for 20 min, respectively. The ultraviolet ozone was used to remove the organic residues of the ITO surface. To fabricate the compact SnO_2_:Ti_3_C_2_ layer, the SnO_2_:Ti_3_C_2_ colloid solution by water to the concentration of 2.14 wt% was spin-coated on ITO substrates at 5000 rpm for 30 s and then annealed at 150 °C for 30 min. The Er^3+^ doped CsPbI_3_ PQDs film was fabricated on the SnO_2_:Ti_3_C_2_ layer by spin-coating at 600 rpm for 6 s and 4000 rpm for 40 s, respectively. The PBDB-TF in chlorobenzene (CB) (400 μL) at various concentrations was dropped on a substrate at 20 s before the end of the spinning process. After that, the PBDB-TF:BTP-4Cl solution was spin-coated on a perovskite layer at 1800 rpm for 60 s and subsequently annealed at 80 °C for 10 min. The Ag electrode was deposited by thermal evaporation to complete the device fabrication. Then, the edge surface of Cr/Ce/Mn-LC with an edge size of 0.1 × 0.04 cm was attached and fixed to the ITO layer of PD with an area of 0.1 × 0.1 cm. When UV light radiation on the face of Cr/Ce/Mn-LC, then the emitted 400–700 nm light is coupled out of the edge surface into the ITO of PDs. The visible and NIR lights directly pass through the ITO and reach the PD. Because the Cr/Ce/Mn-LC only occupies a part of the surface of the ITO layer and has high transparency for photons with a longer wavelength (>410 nm), which would not affect the light collection of PD.

### Characterization

UV/vis-NIR absorption spectra were measured with a Shimadzu UV-3600PC UV/vis-NIR scanning spectrophotometer in the range from 200 to 2500 nm. Patterns were recorded in thin-film mode on a Bruker AXS D8 diffractometer using Cu Kαradiation(λ = 1.54178 Å). Atomic Force Microscope (AFM) was performed using a DI Innova AFM (Bruker) in light tapping mode. The morphology of the products was recorded with a Hitachi H-8100IV transmission electron microscope (TEM) under an acceleration voltage of 200 kV. The samples were pumped using a laser system consisting of a tunable optical parameter oscillator (OPO, Continuum Precision II 8000) with a pulse duration of 10 ns, a repetition frequency of 10 Hz, and a line width of 4–7 cm^−1^. A visible photomultiplier (350–850 nm) combined with a double-grating monochromator were used for spectral collection. The X-ray photoelectron spectroscopy (XPS) was carried out in a Kratos Axis Ultra DLD spectrometer equipped with a monochromatic Al Kα X-ray source (hν = 1486.6 eV) operated at 150 W with a multichannel plate, and a delay line detector under 1.0 × 10^−9^ Torr vacuum. A photomultiplier combined with a monochromator was used for dynamics signal collection of samples from 350 to 850 nm. Nanosecond fluorescence lifetime experiments were performed by the time-correlated single-photon counting system (HORIBA Scientific iHR 320). Absolute photoluminescence quantum yield measurements were performed on colloidal CsPbCl_3_:Cr^3+^,Ce^3+^,Mn^2+^ and CsPbI_3_:Er^3+^ PQDs (dispersed in toluene placed in a sealed 1 cm path length quartz cuvette) and Cr/Ce/Mn-LC. They were positioned in a Teflon-based integrating sphere using a custom cuvette holder and directly excited with a 365 nm Xe lamp. The typical PLQY in such a system is estimated as follows:

$${{{\mathrm{PLQY}}}} = \frac{{N_{{\mathrm{em}}}}}{{N_{{\mathrm{abs}}}}} = \frac{{{\int} {I_{{\mathrm{sample}}}\left( \lambda \right) - I_{{\mathrm{ref}}}\left( \lambda \right)d\lambda } }}{{{\int} {E_{{\mathrm{ref}}}\left( \lambda \right) - E_{sample}\left( \lambda \right)d\lambda } }}$$

where N_em_ and N_abs_ are the numbers of emission and absorption photons of samples. I_sample_ and E_sample_ present the spectral intensity of the emitted light and excitation light of samples, and I_ref_ and E_ref_ is the spectral intensity of the emitted light and excitation light for a reference cuvette containing neat toluene. The monochromatic light was from a Newport Oriel 200TM. The Mott–Schottky curves via capacitance-voltage measurements of CsPbI_3_: Er^3+^ PQDs are obtained by a Princeton electrochemical workstation (Parstat Mc Princeton Instruments Co., Ltd., USA). The Xe lamp (LSB-X150AUV 200–2500 nm, Zolix) with the spectral range from 200 to 2500 nm equipped with a monochromator (Omni-λ3007i, Zolix) was used to generate the monochromatic light to conduct the spectral response measurements. Actually, the intensity of the Xe lamp is weak in the region of 200–300 nm, thus we must correct it before the measurement.

## Supplementary information

supporting information
