# Supplementary material for: A novel approach for designing efficient broadband photodetectors expanding from deep ultraviolet to near infrared
Source: Light Sci Appl. 2022 Apr 11;11:91. doi: 10.1038/s41377-022-00777-w (PMC9001727; doi:10.1038/s41377-022-00777-w)
Supplement: Supplementary file 1 — supporting information [file 41377_2022_777_MOESM1_ESM.pdf]

Supplementary Information for

A novel approach for designing efficient broadband photodetectors expanding from deep ultraviolet to near infrared

Nan Ding,[1#] Yanjie Wu,[1#] Wen Xu,[1, 2] Jiekai Lyu,[1] Yue Wang,[1] Lu Zi,[1] Long Shao,[1] Rui Sun,[1] Nan Wang,[1] Sen Liu,[1] Donglei Zhou,[1] Xue Bai,[1] Ji Zhou,[3] Hongwei Song*[1]*

[1] State Key Laboratory on Integrated Optoelectronics, College of Electronic Science and Engineering, Jilin University, Changchun, 130012, China.

[2] Key Laboratory of New Energy and Rare Earth Resource Utilization of State Ethnic Affairs Commission, Dalian Minzu University, Dalian 116600, China

[3] State Kay Lab of New Ceramics and Fine Processing, Department of Materials Science and Engineering, Tsinghua University, Beijing 100084, China.

[*]E-mail: Prof. Wen Xu (xuwen@dlnu.edu.cn), Prof. Hongwei Song (songhw@jlu.edu.cn).

Supplementary Note 1

According to previous reports [1], the increase of the band gap of PQDs could be divided into two reasons: (1) the quantum confinement effect; (2) the decrease of the lattice constant. The contribution of the quantum confinement effect is calculated as follows [2]:

$$\Delta E = \frac{\hbar^2 \pi^2}{2 m_r R^2} - \frac{1.786 e^2}{4 \pi \varepsilon_0 \varepsilon R}$$

where R represent the particle radius, m_r denotes the effective mass of the exciton, ε_0 and ε are the vacuum permittivity and the relative dielectric constant of $CsPbI_3$ bulk material. According to the equation, the calculated blue-shift of the band gap for $CsPbI_3$ PQDs within 13 meV with the particle size decrease from 11.4 nm to 10.3 nm. Compared to the experimental results (~70 meV), the decrease of lattice constant of PQDs after doping should be dominated.

Supplementary Note 2

The defect density (N_t) and the carrier mobility (μ) of $CsPbI_3$: Er^{3+} PQDs are obtained by dark current-voltage measurement technique in ITO/SnO_2/ PQDs /Ag devices, as displayed in Figure

S8. They can be estimated as follows [3]:

$$N_t = \frac{2\varepsilon_0\varepsilon V_{TFL}}{eL^2}$$

$$\mu = \frac{8JL^3}{9\varepsilon\varepsilon_0 V^2}$$

where V_{TFL} is the trap-filled limit voltage, L is the thickness of the PQDs film, ε_0 and ε are the vacuum permittivity and the relative dielectric constant of $CsPbI_3$ bulk material, J and V are the current density and the applied voltage.

Supplementary Note 3

The conductivity (σ) of PQDs are obtained in ITO/ PQDs /Ag devices. They can be estimated as follows [4]:

$$I = \sigma AD^{-1}V$$

where A is the contact area, D is the thickness of the PQDs films, V represents the applied voltage.

Supplementary Note 4

The Mott–Schottky curves via capacitance-voltage measurements of $CsPbI_3$: Er^{3+} PQDs are obtained in ITO/SnO_2/ PQDs /Ag devices. They can be estimated as follows [5]:

$$\frac{1}{C^2} = \frac{2}{A^2\varepsilon_0\varepsilon eN}(V_b - V)$$

where C is the measured capacitance, A is the active area, V is the bias, ε is the static permittivity, ε_0 is the permittivity of free space, e is the elementary charge, and N is the doping density of the donor that represents carrier density in PDs.

The built-in potential (V_b) values can be extracted based on the Mott-Schottky equation. A larger built-in potential value means an enhanced driving force for the separation of photogenerated carriers as well as an extended depletion region for efficient suppression of electron-hole recombination, which is favorable for the carrier separation, transport, and extraction.[6-7]

Supplementary Note 5

Structural optimization, the formation energy of structure and bonding energy:

The time-averaged cubic $CsPbI_3$ is refined to Pm-3m (227) global symmetry with 1fu/cell.[8] We relaxed the lattice constant of cubic (Pm-3m) of $CsPbI_3$ and $CsErI_3$ by keeping the global symmetry (Pm-3m) with 12×12×12 k-grid. However, the theoretically predicted cubic structure of halide perovskites does not have perfect Wyckoff positions presented in cubic Pm-3m structure.[9] Therefore, we used 2×2×2 supercell of cubic Pm-3m structure of cubic $CsPbI_3$ as initial structure, and then fully relaxed the internal atomic positions by using 6x6x6 k-mesh. The 12.5% doped cubic $CsPbI_3$ with formula $Cs_8ErPb_7I_{24}$ was constructed based on relaxed 2×2×2 supercell of cubic $CsPbI_3$ by replacing one Pb by Er, then keeping cubic out-shape and relaxed in internal position and lattice constants. Both the relaxed 2×2×2 cubic supercell are with symmetry breaking with respect to Pm-3m global symmetry. The bond energy of Er-I and Pb-I bond in cubic $CsErI_3$ and $CsPbI_3$ is roughly estimated by using cubic (Pm-3m) structure. While the relaxed 2×2×2 supercells of cubic $CsErI_3$ and $CsPbI_3$ were used to achieve formation energy to binary compounds.

Formation energy of intrinsic defects:

The formation energy of defects can be predicted as expressed in the following equation:

$$\Delta H_{D,q}(E_F,\mu) = \left[E_{D,q} - E_H\right] + \sum_i n_i\mu_i + q_iE_F + E_{corr} \qquad (1)$$

where $E_{D,q}$ and E_H are the total DFT internal energies of the defect supercell and host supercell, respectively. μ_i refers to the chemical potential of the atom i (host or impurity) added ($n_i < 0$) or removed ($n_i > 0$) from the host supercell to form the defect. q_i is the charge state of the defect. E_F is the Fermi energy, and E_{corr} is the term that accounts for the finite-size corrections, within the supercell approach.

In this work, we explored the intrinsic vacancy defects of Cs, Pb and I in supercell (64 fu/cell) of cubic $CsPbI_3$ and Er-doped cubic $CsPbI_3$. The host supercells were constructed by using the cubic $CsPbI_3$ and Er-doped $CsPbI_3$ with internal symmetry breaking. Instead of considering all possible defect sites, we selected the sites near doped Er atoms, to explore the coupling between Er and vacancy defects. Then by freezing the cubic lattice constants, we fully relaxed the internal atomic positions in the defect and host supercell without considering global symmetry with Gamma-only k-grid. Next, we achieved the total energy of the host and defect supercell structure by using 2x2x2 k-grid for calculating the formation energy of defects. Note that the E_{corr} term is

not considered in our calculation because we suggested that the finite-size effect is negligible within 64 fu/cell.

Stability of Er^{3+} doped $CsPbI_3$:

In our work, considering the different phase structure between $Cs_3Er_2I_9$ (hexagonal) and $CsPbI_3$ (cubic), it is improbable to directly theoretically compare the physical stability. Meanwhile, when doping a small amount of Er^{3+}, the cubic $CsPbI_3$: Er^{3+} is obtained with occupying the lattice of Pb^{2+} by Er^{3+}. Thus, we proposed to compare the stability between the cubic $CsErI_3$ ($[CsEr^{3+}I_3]$+e) and $CsPbI_3$ with the same phase structure. Similar comparison methods have also been used in previous literatures.[10-11]

Supplementary Note 6

The detectivity (D*) of the device can be estimated as follows [12]:

$$D^* = (S\Delta f)^{1/2}R/J_d$$

where S, Δf, R and J_d present the effective area, the electrical bandwidth, the responsivity and the noise current of the detector. Many sources can contribute to the noise current, including thermal, shot, flicker (1/f) and generation-recombination noise. We note that the noise current cannot be directly inferred from the shot noise (simply defined by the dark current) and thermal noise in disordered semiconducting systems. In organic semiconductors (eg., PDDTT, $PC_{60}BM$, PTPD) and colloidal quantum-dot photodetectors (eg., PbS, HgTe, HgSe), the actual measured noise can often exceed the root mean square of shot and thermal noises.[13] In our work, the shot noise from the dark current (i_n) is the major contributor to the overall photodetector noise. [14,15] Therefore, the *D** can be expressed as: $D^* = \sqrt{\frac{S}{2eI_d}}R$, where e is the absolute value of electron charge and I_d is the dark current.

Supplementary Note 7

Modelling of optical quantum efficiencies of Cr/Ce/Mn-LC:

We adopted the analytic model developed for calculating the optical quantum efficiencies

(external and internal) of Cr/Ce/Mn-LC. [16] Following the previous procedure, we obtain:

$$\eta_{int} = \eta_{col} = \frac{\eta_{PL} * \eta_{trap}}{1+\beta(\alpha_2+S)*l*(1-\eta_{PL}*\eta_{trap})}$$

where η_{int} or η_{col} is the ratio between edge emitted photons and absorbed photons, α_2 is the absorption coefficient of the LC fluorophores at the emission wavelength, S is the scattering coefficient, β is a constant (~1.4), *l* is the LC length, η_{PL} is the PLQY of PQDs measured in dilute solutions, and η_{trap} of ~75% is determined by the refractive index of the waveguide [~1.5 for common glass materials and polymers such as poly(methyl methacrylate)] and that of the air (~1). In the case of zero reabsorption ($\alpha_2 = 0$), scattering becomes the efficiency-limiting factor. This situation is realized in our Cr/Ce/Mn-LC, where we have:

$$\eta_{int} = \eta_{col} = \frac{\eta_{PL} * \eta_{trap}}{1+\beta*S*l*(1-\eta_{PL}*\eta_{trap})}$$

The PLQY of $CsPbCl_3$: Cr^{3+}, Mn^{2+}, Ce^{3+} PQDs in the PMMA film reaches 81.5 %, enabling a LC with an η_{int} of 61.13 %. The ratio between face emitted photons and absorbed photons can be lowed than 20.37%.

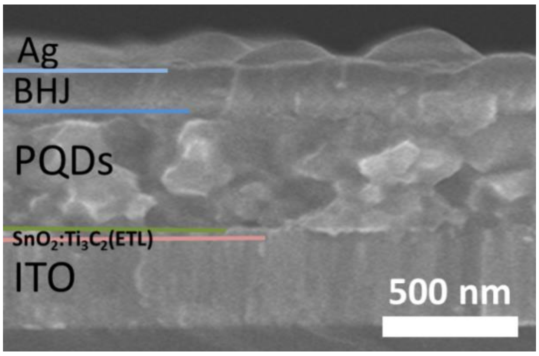

Figure S1. Cross-sectional SEM image of the broadband PDs.

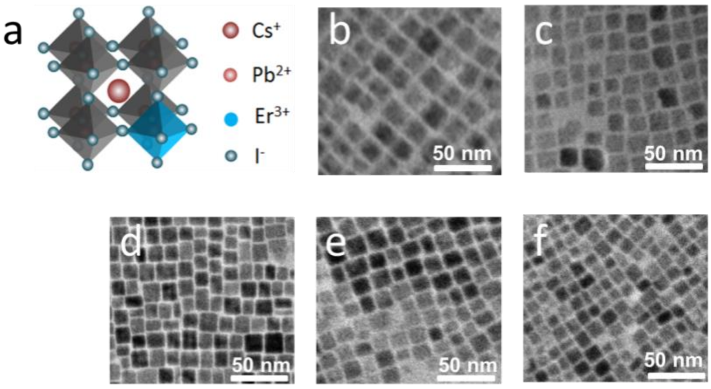

Figure S2. (a) Structure of $CsPbI_3:Er^{3+}$ PQDs; TEM images for $CsPbI_3:Er^{3+}$ PQDs with different doping concentrations (b-f : 0%, 1.2%, 4.6%, 7.7%, 9.7%).

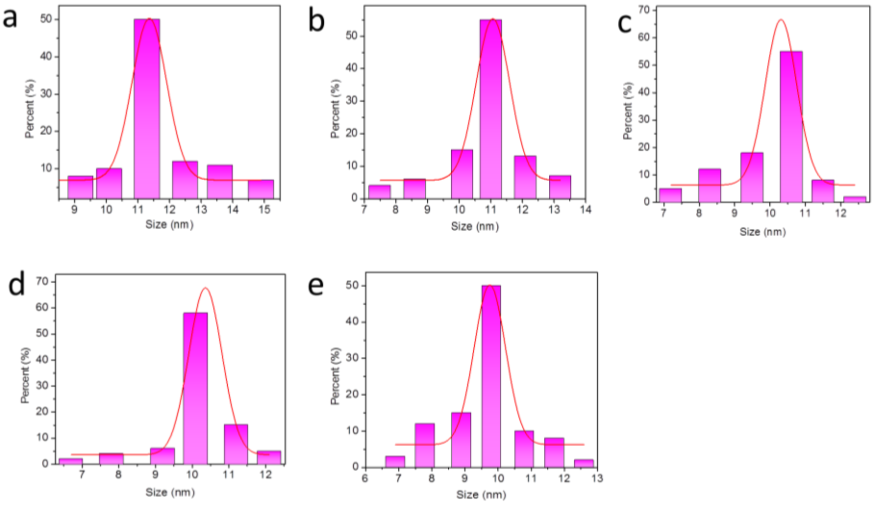

Figure S3. Size distributions Er^{3+} ions doped $CsPbI_3$ PQDs (a-e: 0%, 1.2%, 4.6%, 7.7%, 9.7%).

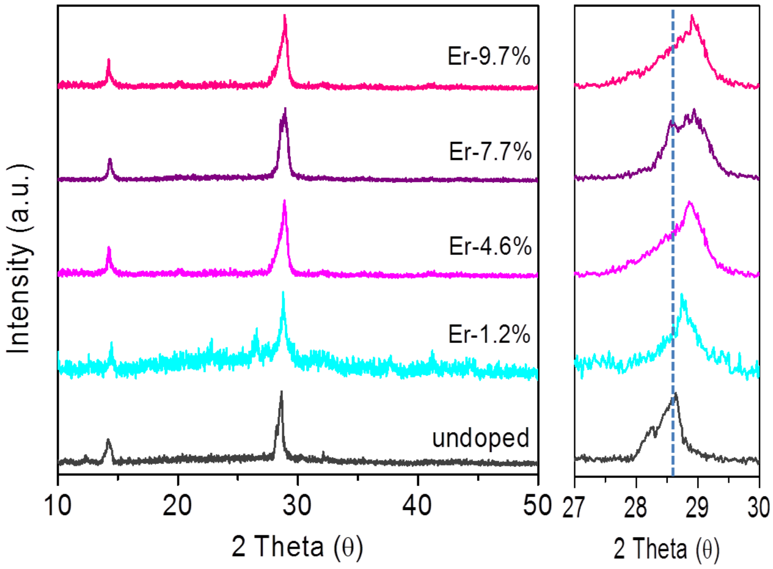

Figure S4. XRD patterns of $CsPbI_3:Er^{3+}$ PQDs with different Er^{3+} concentrations of 0%, 1.2%, 4.6%, 7.7%, 9.7%.

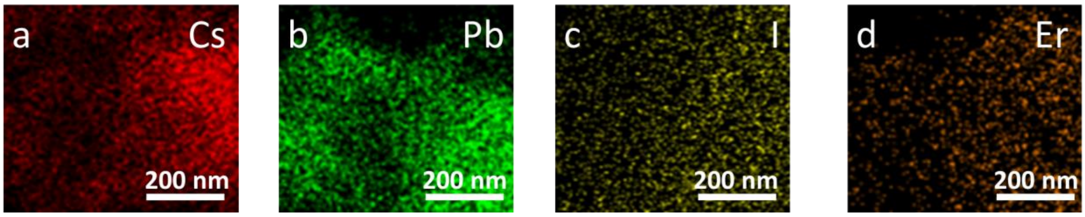

Figure S5. Energy-dispersive X-ray mapping images of $CsPbI_3:Er^{3+}$ (7.7%) PQDs.

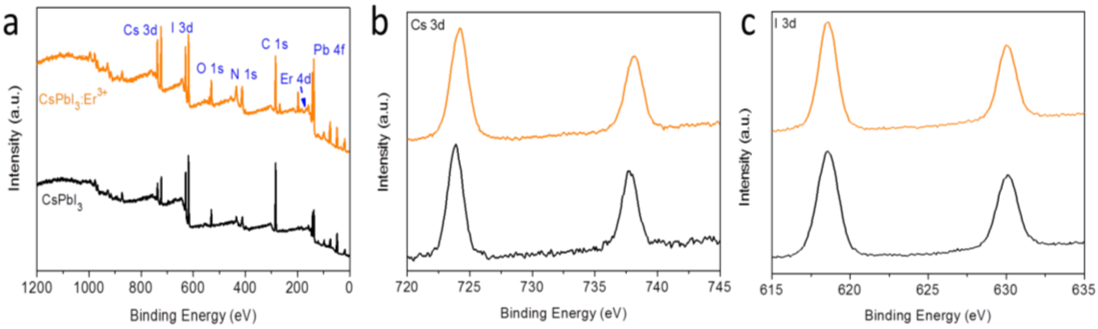

Figure S6. (a) XPS spectra of $CsPbI_3$ and $CsPbI_3:Er^{3+}$ (7.7%) PQDs, (b-c) Corresponding high-resolution XPS spectra of Cs 3*d* and I 3*d*.

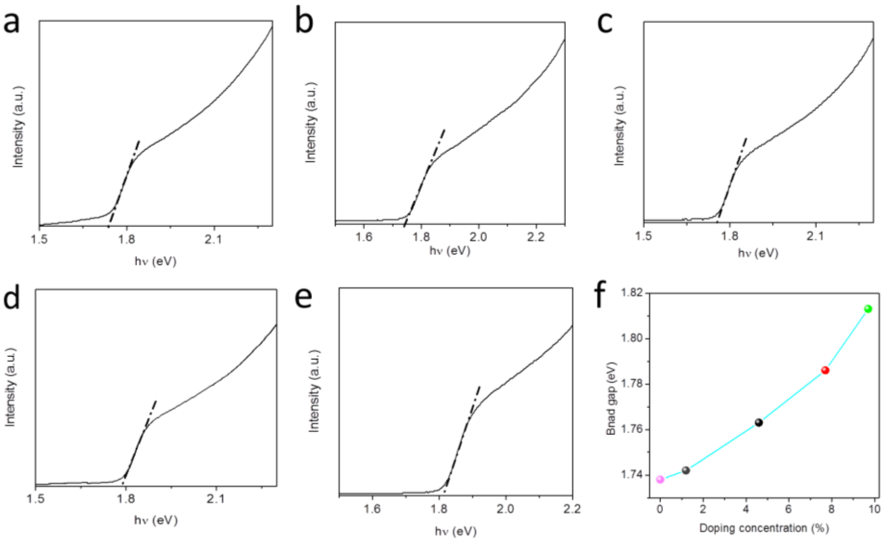

Figure S7. Tauc plots of $CsPbI_3:Er^{3+}$ PQDs with different Er^{3+} concentrations (a-e: 0%, 1.2%, 4.6%, 7.7%, 9.7%).

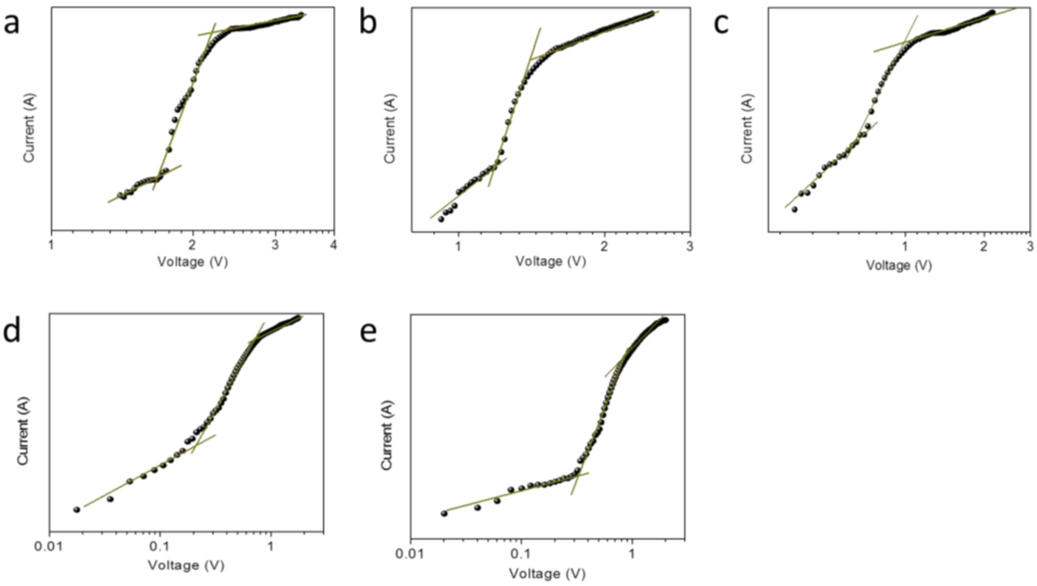

Figure S8. Trap density extraction by dark current-voltage measurement of the electron-only device of Er^{3+} doped $CsPbI_3$ PQDs with different concentrations (a-e: 0%, 1.2%, 4.6%, 7.7%, 9.7%), with the device structure of ITO/SnO_2/PQDs /Ag.

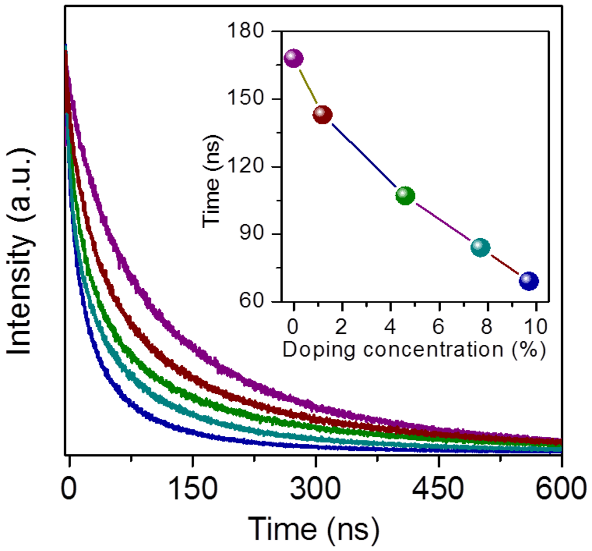

Figure S9. Time-resolved PL decay lifetimes of Er^{3+} doped $CsPbI_3$ PQDs with different concentrations (0%, 1.2%, 4.6%, 7.7%, 9.7%).

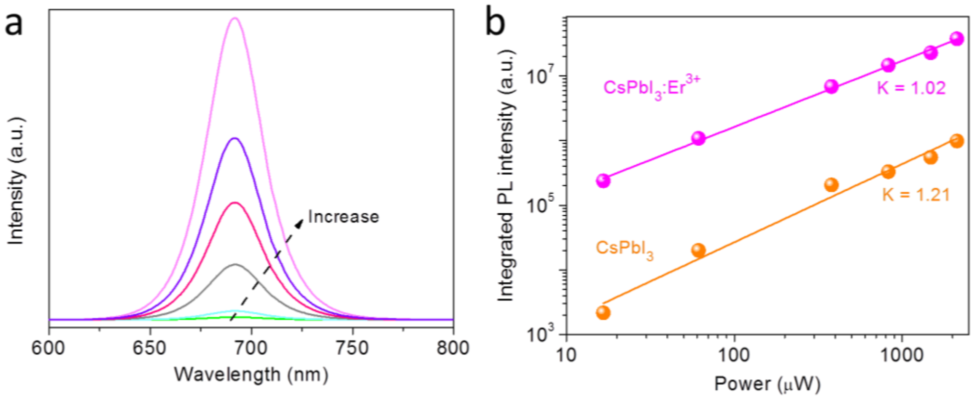

Figure S10. (a) Steady-state PL spectra of $CsPbI_3:Er^{3+}$ (7.7%) PQDs with the excitation power density from 16.6-2125 μW, and (b) Logarithm plot of the integrated PL intensity versus excitation power density of $CsPbI_3$ and $CsPbI_3:Er^{3+}$ (7.7%) PQDs.

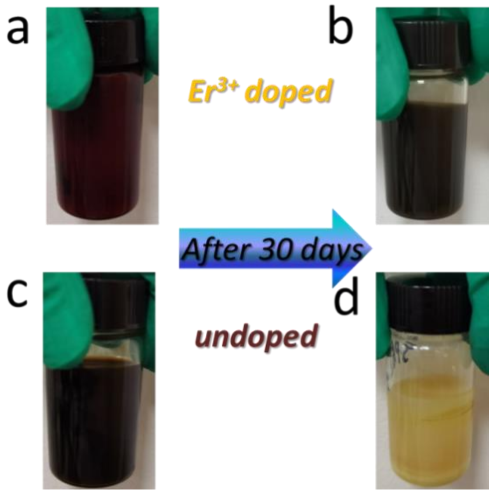

Figure S11. Photos of $CsPbI_3$ and $CsPbI_3:Er^{3+}$ (7.7 %) PQDs after 30 days under sunlight.

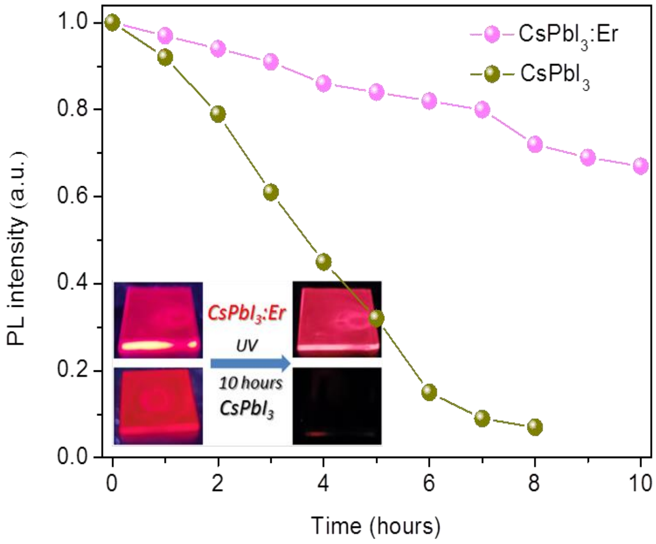

Figure S12. Normalized PL intensity of $CsPbI_3$ and $CsPbI_3:Er^{3+}$ (7.7 %) PQDs as a function of UV light radiation time.

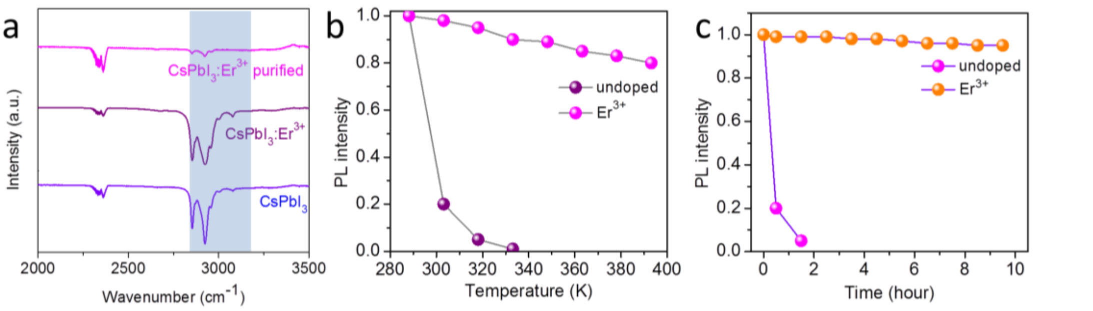

Figure S13. (a) Fourier Transform Infrared spectra of $CsPbI_3$ and $CsPbI_3$:Er^{3+} (7.7 %) PQDs treated with ethyl acetate, (b-c) PL intensity of $CsPbI_3$ and $CsPbI_3$:Er^{3+} (7.7 %) PQDs as a function of temperature and time.

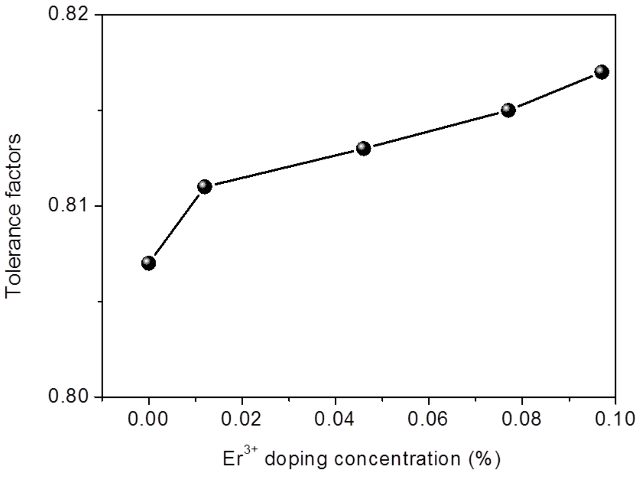

Figure S14. Tolerance factors of $CsPbI_3$:Er^{3+} PQDs with different Er^{3+} concentrations (0%, 1.2%, 4.6%, 7.7%, 9.7%)

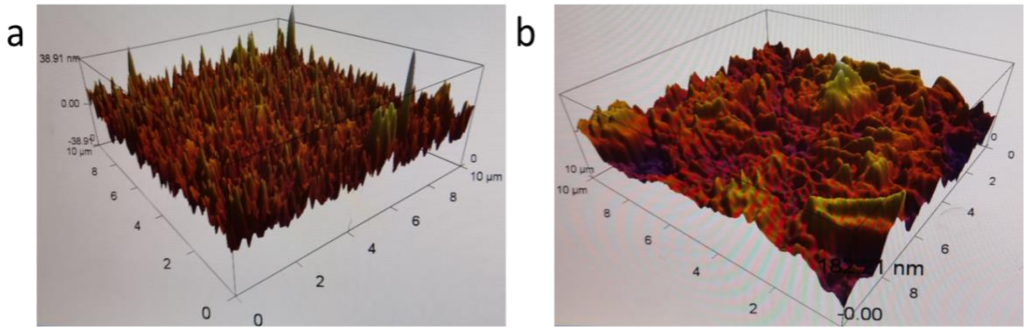

Figure S15. AFM 3D images of $CsPbI_3$:Er^{3+} (7.7 %) PQDs film and $CsPbI_3$:Er^{3+} (7.7 %) PQDs /BHJ film.

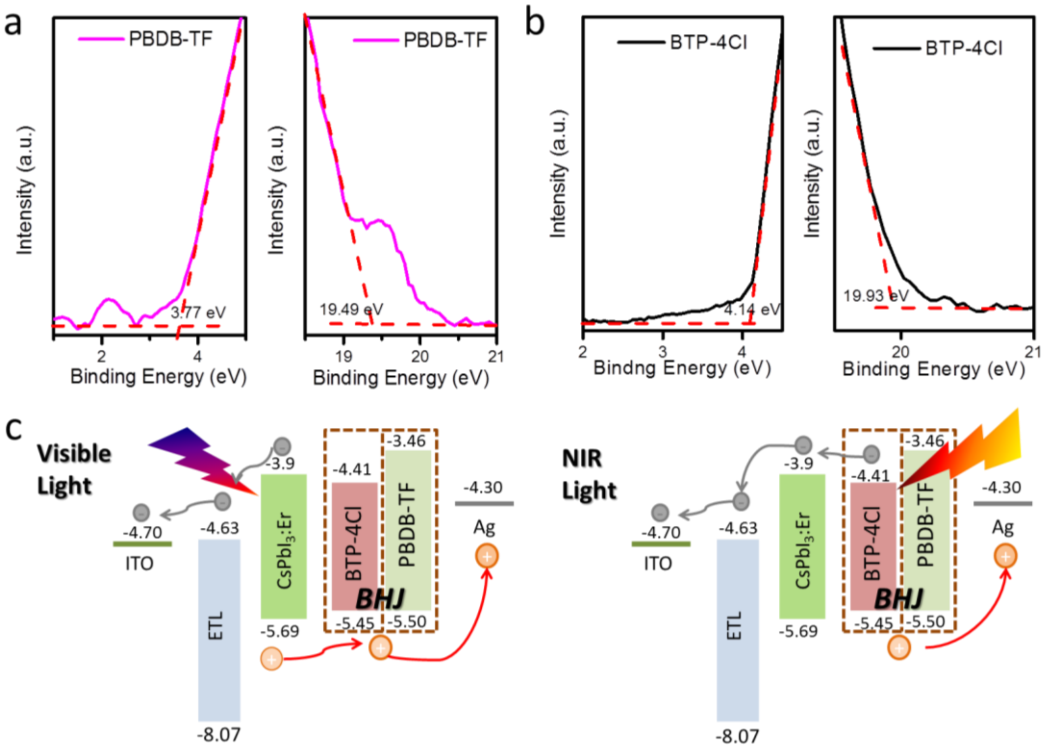

Figure S16. (a-b) The UPS curves of PBDB-TF and BTP-4Cl films. (c) Energy band schematic visible light and NIR light induced charge transfer of heterojunction based on $CsPbI_3:Er^{3+}$ PQDs and BHJ film.

The proposed charge generation and transport mechanism of this heterojunction device is shown in Figure S16, which is designed for the following reasons: (1) The $CsPbI_3:Er^{3+}$ PQDs is a direct band gap semiconductor and the exciton can be easily separated due to its weak binding energy;[17] (2) The PQDs film has high electron and hole mobility, showing minimal charge recombination probability;[18] (3) Upon visible light illumination, the BHJ film can be a useful hole transport channel, thus holes generated in the PQDs film can transport through the BHJ and collect Ag anode; (4) Similarly, upon NIR light illumination, the electron that is generated from the donor-acceptor interface in the BHJ film can transport to the PQDs film and be collected by SnO_2/ITO cathode under a local electric field, due to the high electron mobility of PQDs film.

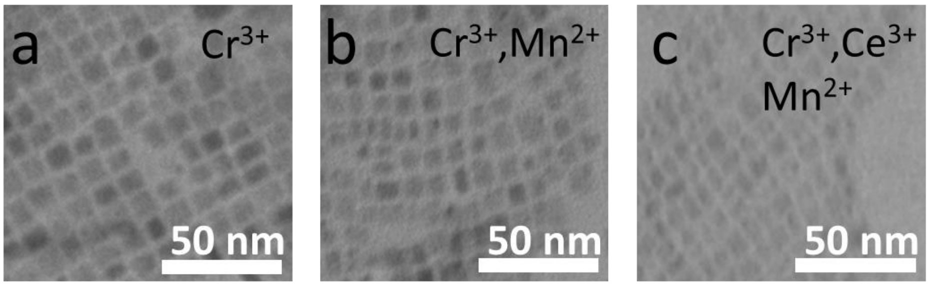

Figure S17. TEM images of Cr^{3+}, Cr^{3+}/Mn^{2+}, and $Cr^{3+}/Mn^{2+}/Ce^{3+}$ doped $CsPbCl_3$ PQDs.

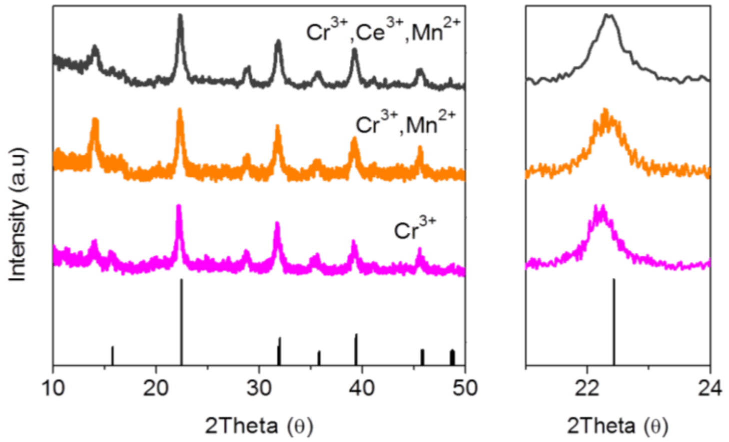

Figure S18. XRD patterns of Cr^{3+}, Cr^{3+}/Mn^{2+}, and $Cr^{3+}/Mn^{2+}/Ce^{3+}$ doped $CsPbCl_3$ PQDs.

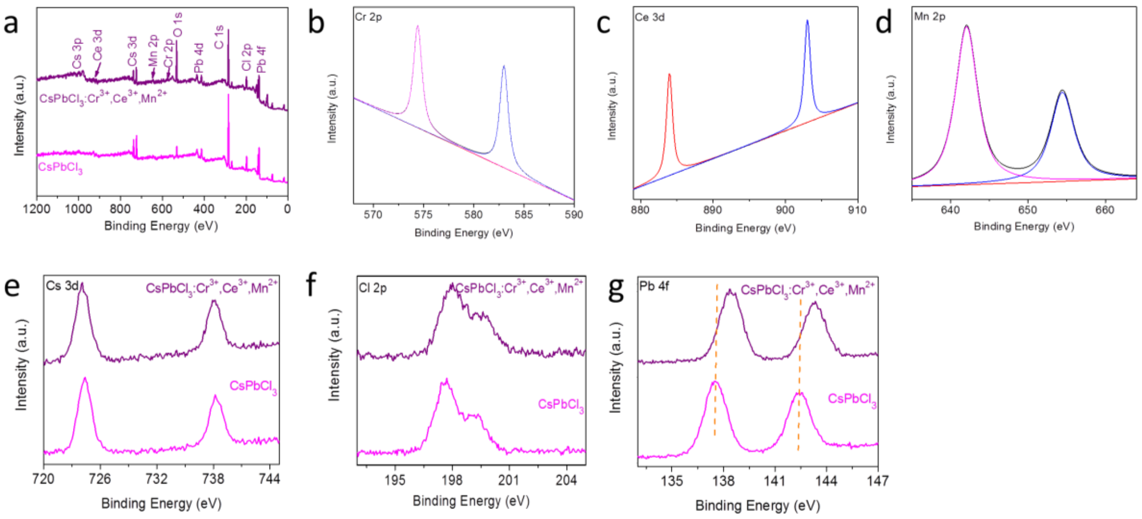

Figure S19. (a) XPS spectra of $CsPbCl_3$ PQDs and $Cr^{3+}/Mn^{2+}/Ce^{3+}$ doped $CsPbCl_3$ PQDs, (b-g) Corresponding high-resolution XPS analysis of Cs 3*d*, Cl 2*p*, Pb 4*f*, Cr 2*p*, Ce 3*d*, and Mn 2*p*， respectively.

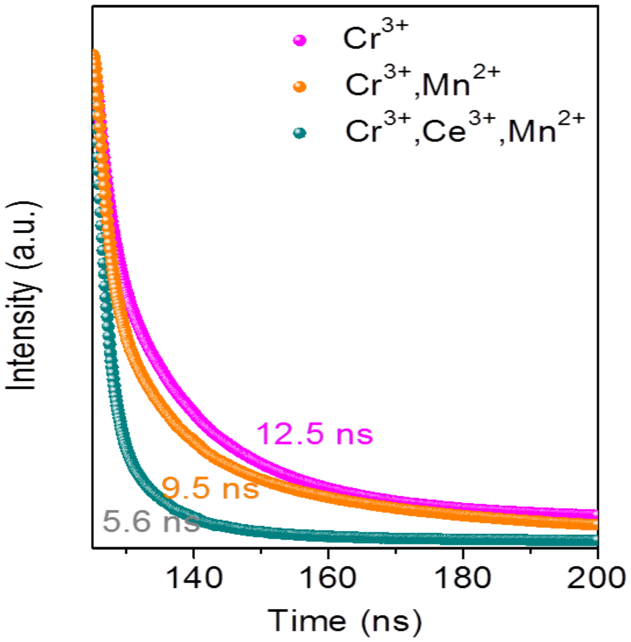

Figure S20. Decay lifetime constants of excition emission in $CsPbCl_3$:Cr^{3+} PQDs after further doping with Mn^{2+} and Mn^{2+}/ Ce^{3+}.

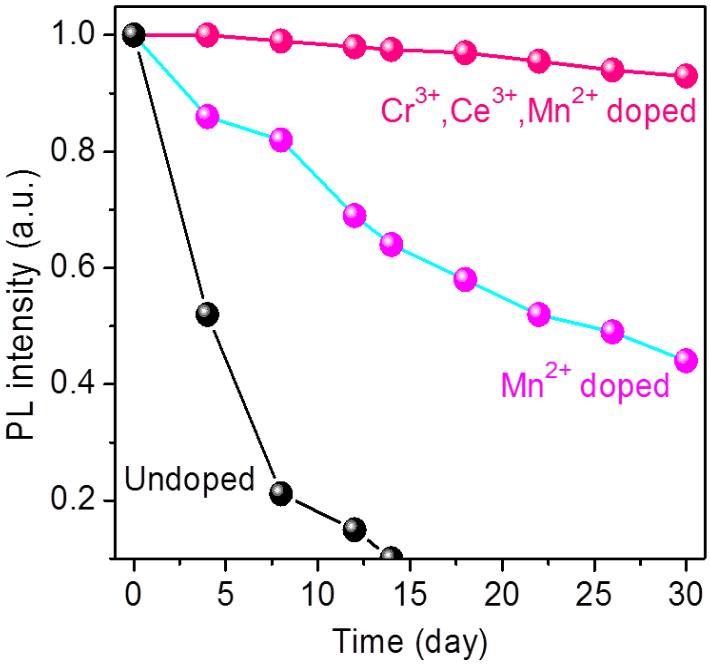

Figure S21. Stability of undoped, Mn^{2+}, and Cr^{3+}/Mn^{2+}/Ce^{3+} doped $CsPbCl_3$ PQDs.

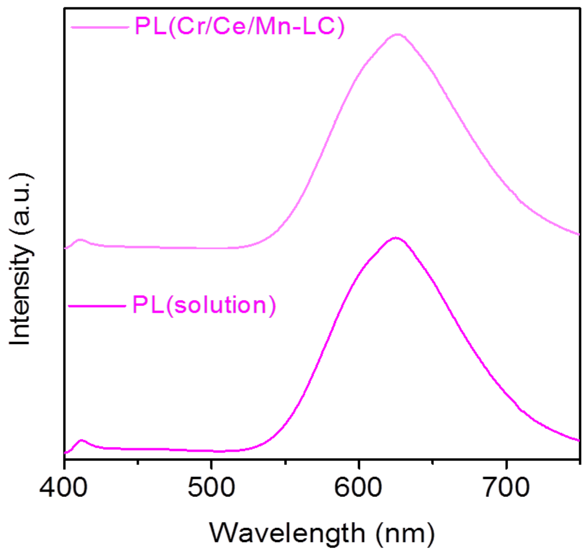

Figure S22. PL spectra of Cr^{3+}-Mn^{2+}-Ce^{3+} doped $CsPbCl_3$ PQDs in solution and LC.

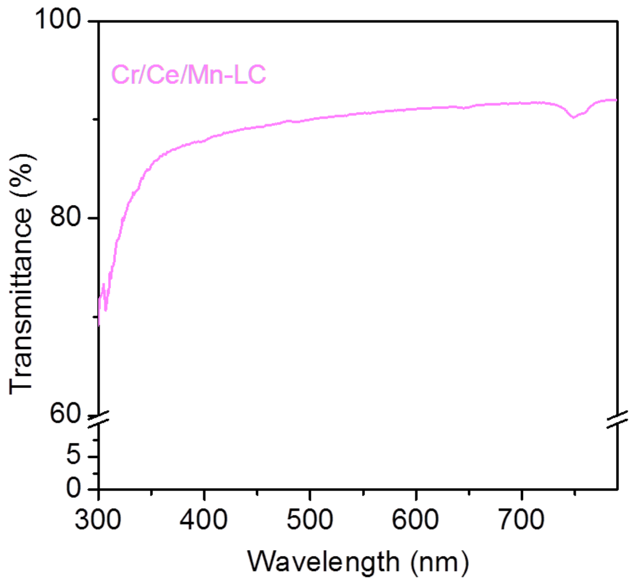

Figure S23. Transmittance spectrum of Cr/Mn/Ce-LC.

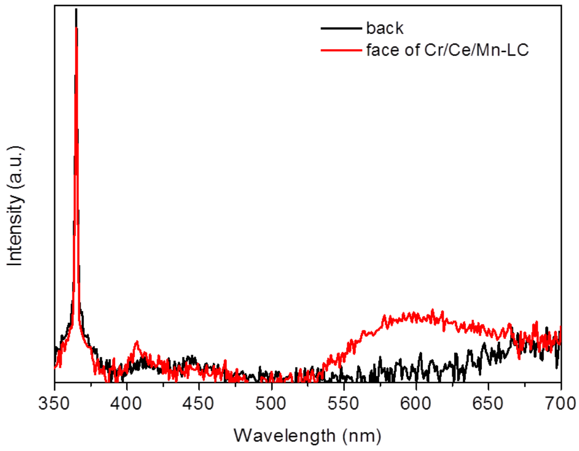

Figure S24. The PLQY of the face of Cr/Ce/Mn-LC and the other edges were fully covered by Al films.

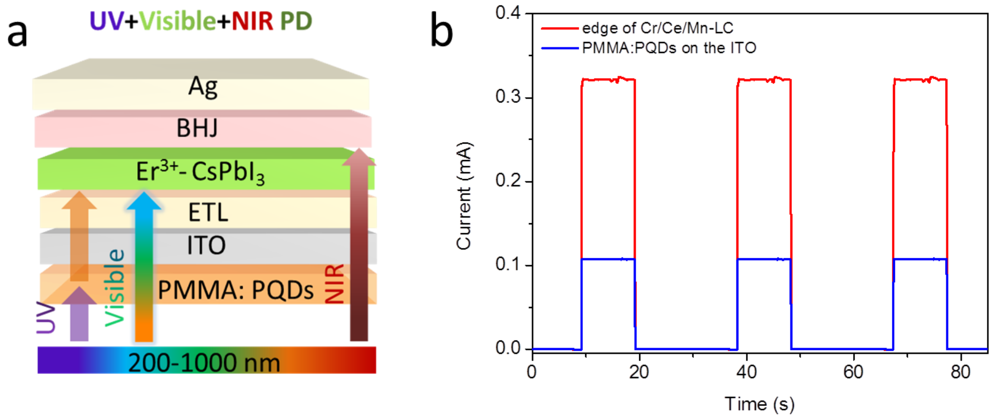

Figure S25. (a) Schematic device structure a broadband PD with the PMMA: $CsPbCl_3:Cr^{3+},Mn^{2+},Ce^{3+}$ PQDs on the ITO. (b) Photocurrents of the edge of Cr/Ce/Mn-LC / ITO / ETL / $CsPbI_3:Er^{3+}$ (7.7 %) / Ag and the PMMA: $CsPbCl_3:Cr^{3+},Mn^{2+},Ce^{3+}$ PQDs / ITO / ETL / $CsPbI_3:Er^{3+}$ (7.7 %) / BHJ / Ag devices under the 260 nm.

It can be seen that the photocurrent of PMMA: $CsPbCl_3:Cr^{3+},Mn^{2+},Ce^{3+}$ PQDs on the ITO is 0.105 mA, which increases to 0.32 mA for the side edge of S4 PDs.

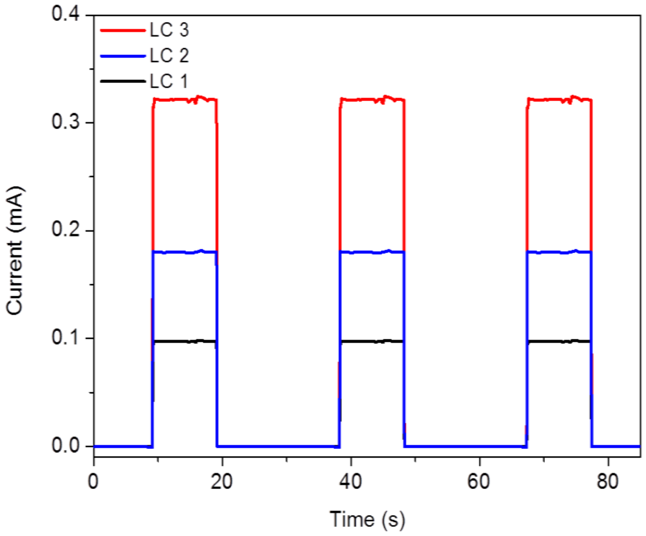

Figure S26. Photocurrents of the surface of Cr/Ce/Mn-LC (LC 1), the edge of Cr/Ce/Mn-LC (LC 2), the edge of Cr/Ce/Mn-LC and the other edges are fully covered by Al films (LC 3) devices under the 460 nm, respectively.

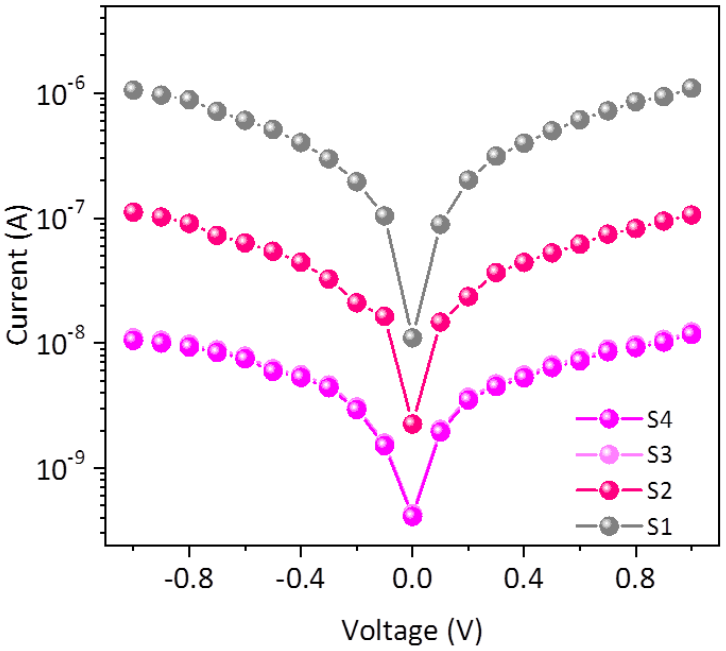

Figure S27. Current density–voltage (I–V) characteristics of S1-S4 under the dark conditions.

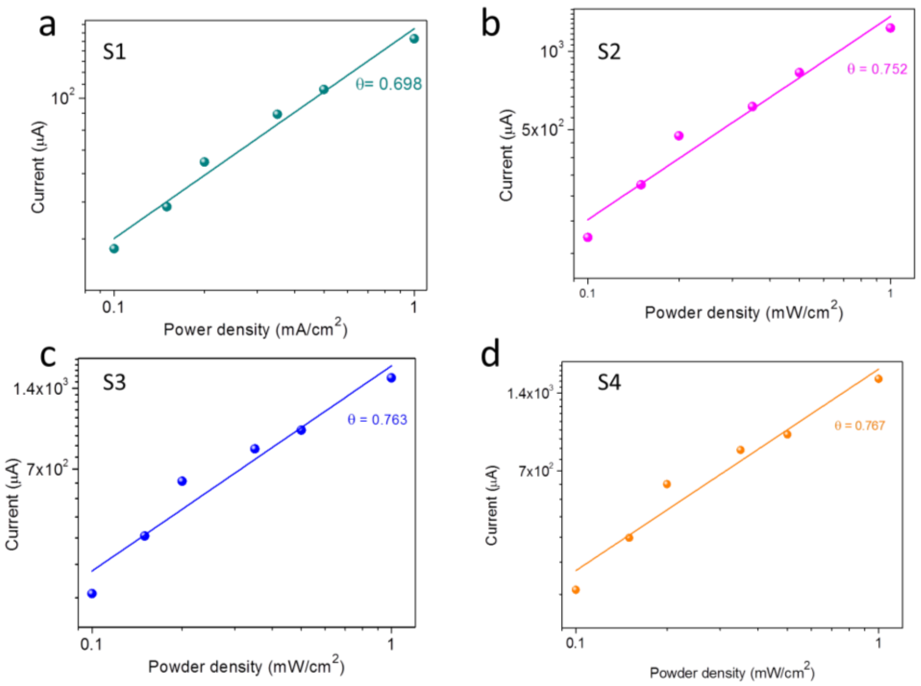

Figure S28. Relationship between current and incident light (460 nm) intensity for the S1-S4 PDs.

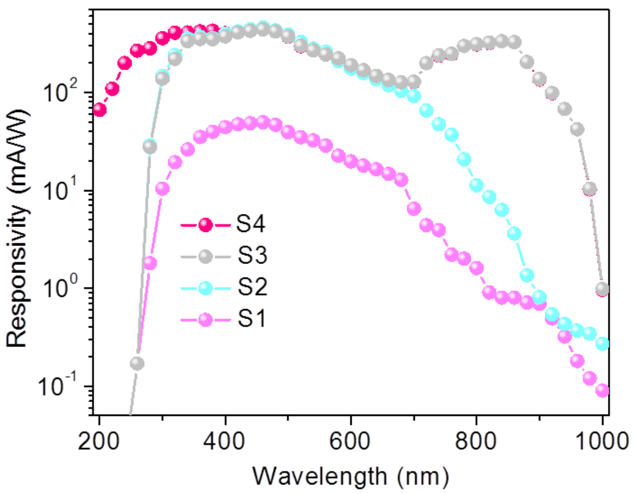

Figure S29. R of the S1-S4 devices.

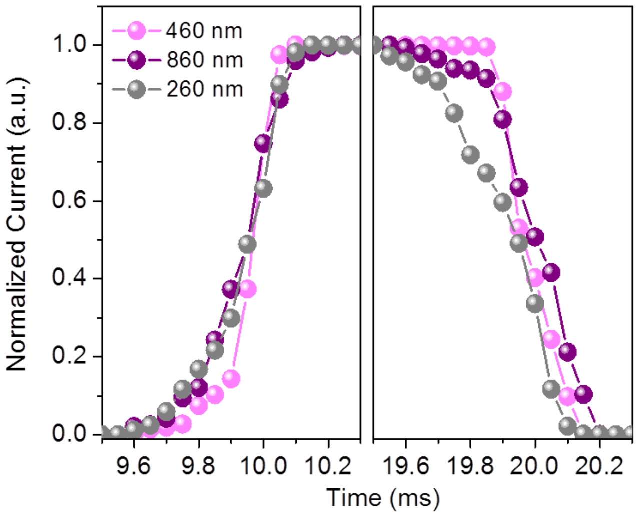

Figure S30. Response time of the S4 PDs under the illumination of 260 nm, 460 nm, and 860 nm, respectively.

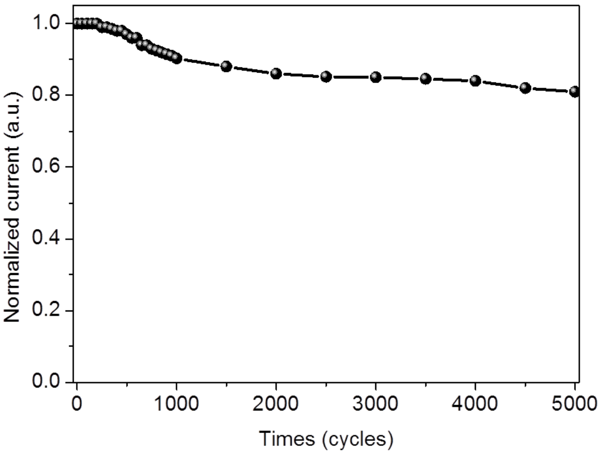

Figure S31. Different on/off cycles of the S4 PDs under the illumination of 460 nm.

Table S1. ICP-OES analysis of $CsPbI_3:Er^{3+}$ PQDs with different molar ratios of ErI_3 to PbI_2 in the synthesis.

Samples	$ErI_3:PbI_2$	Molar ratio (Er:Pb)
Er-1.2%	0.5:1	0.012:1
Er-4.6%	1:1	0.046:1
Er-7.7%	1.5:1	0.077:1
Er-9.7%	2:1	0.097:1

Table S2. PLQY, decay timeconstants (τ), radiative decay rate (k_r), nonradiative decay rate (k_{nr}) of $CsPbI_3$ and $CsPbI_3:Er^{3+}$ PQDs as a function of Er^{3+} doping concentration.

PQDs	PLQY (%)	τ (ns)	K_r ($*10^6 S^{-1}$)	K_{nr} ($*10^6 S^{-1}$)
**$CsPbI_3$**	**52**	**168**	**3.1**	**2.9**
**$CsPbI_3:Er^{3+}$(1.2%)**	**63**	**131**	**4.81**	**2.82**
**$CsPbI_3:Er^{3+}$(4.6%)**	**72**	**107**	**6.73**	**2.62**
**$CsPbI_3:Er^{3+}$(7.7%)**	**93**	**84**	**11.1**	**0.83**
**$CsPbI_3:Er^{3+}$(9.7%)**	**82**	**69**	**11.9**	**2.6**

Table S3. Defects in cubic $CsPbI_3$ at u_{rich}(Pb) and u_{poor}(I).

	Charge q	E_{Defect} (eV)	E_{Host} (eV)	δE (eV)	Note
V_{Cs}	0	-974.56135385	-979.10389559	4.5425	u_{Pb}=0.0 eV<br>u_{Cs}= -2.11 eV<br>u_I= -0.97 eV<br>E_{VBM}=1.8899 eV
V_{Cs}	-1	-972.69781145	-979.10389559	6.4061	
V_{Pb}	0	-972.62504398	-979.10389559	6.4789	
V_{Pb}	-1	-970.92913635	-979.10389559	8.1748	
V_{Pb}	-2	-969.10180146	-979.10389559	10.0021	
V_I	0	-975.12589466	-979.10389559	3.9780	
V_I	1	-978.29055439	-979.10389559	0.8133	

Table S4. Defects in cubic $CsPb_{0.923}Er_{0.077}I_3$, calculated by using Er_2 pseudopotential.

	Charge q	E_{Defect} (eV)	E_{Host} (eV)	E_{Defect} - E_{Host}(eV)	Note
V_{Cs}	0	-967.50142	-975.35466	7.8532	u_{Pb}=0.0 eV<br>u_{Cs}= -2.11 eV<br>u_I= -0.97 eV<br>E_{VBM}=2.0928 eV
V_{Cs}	-1	-965.76569	-975.35466	9.5890	
V_{Pb}	0	-967.81339	-975.35466	7.5412	
V_{Pb}	-1	-967.39736	-975.35466	7.9573	
V_{Pb}	-2	-965.69591	-975.35466	9.6588	
V_I	0	-967.96305	-975.35466	7.3916	
V_I	1	-974.31491	-975.35466	1.0398	

Reference:

1. Zhou, D. L. et al. Impact of host composition, codoping, or tridoping on quantum cutting emission of ytterbium in halide perovskite quantum dots and solar cell applications. *Nano Letters,* **19**, 6904-6913 (2019).

2. Brus, L. E. Electron–electron and electron-hole interactions in small semiconductor crystallites: The size dependence of the lowest excited electronic state. *The Journal of chemical physics,* **80**, 4403 (1984).

3. Yang, D. et al. Surface optimization to eliminate hysteresis for record efficiency planar perovskite solar cells. *Energy & Environmental Science,* **9**, 3071-3078 (2016).

4. Chen, Y. et al. Design of an inorganic mesoporous hole‐transporting layer for highly efficient and stable inverted perovskite solar cells. *Advanced Materials,* **30**, 1805660 (2018).

5. K. K. N. S. M. Sze, Physics of Semiconductor Devices, Wiley, 2006.

6. Laban, W. A. et al. Depleted hole conductor-free lead halide iodide heterojunction solar cells. *Energy & Environmental Science,* **6**, 3249 (2013).

7. Jang, Y. W. et al. Intact 2D/3D halide junction perovskite solar cells via solid-phase in-plane growth. *Nature Energy,* **6**, 63 (2021).

8. Protesescu, L. *et al*. Nanocrystals of cesium lead halide perovskites ($CsPbX_3$, X = Cl, Br, and I): novel optoelectronic materials showing bright emission with wide color gamut. *Nano Letters,* **15**, 3692-3696 (2015).

9. Becker, M. A. *et al*. Bright triplet excitons in caesium lead halide perovskites. *Nature ,* **553**, 189 (2018).

10. Tan, Z. k. *et al*. Highly efficient blue‐emitting bi‐doped Cs2SnCl6 perovskite variant: photoluminescence induced by impurity doping. *Advanced Functional Materials* **28**, 1801131 (2018).

11. Bi, C. H. *et al*. Thermally stable copper(II)-doped cesium lead halide perovskite quantum dots with strong blue emission. *The Journal of Physical Chemistry Letters* **10**, 943-952 (2019).

12. Gong, X. *et al*. High-detectivity polymer photodetectors with spectral response from 300 nm to 1450 nm. *Science* **325**,1665 (2009).

13. Arquer, F,P,G. *et al*. Solution-processed semiconductors for next-generation photodetectors. *Nature Reviews Materials* **2**, 16100 (2017).

14. Zhao, X. H. *et al*. Vertically stacked PEDOT:PSS/PbS/$CsPbCl_3$ for flexible optoelectronic devices. *Journal of Alloys and Compounds* **866**, 158997 (2021).

15. Kim, W. *et al*. Perovskite multifunctional logic gates via bipolar photoresponse of single photodetector. *Nature communications.* **13**, 720 (2022).

16. Wu, K. F., Li, H. B. & Klimov, V. I. Tandem luminescent solar concentrators based on engineered quantum dots. *Nature Photonics* **12**, 105-110 (2018).

17. Ponseca, C. S. *et al*. Organometal halide perovskite solar cell materials rationalized: ultrafast charge generation, high and microsecond-long balanced mobilities, and slow recombination. *Journal of the American Chemical Society,* **136**, 5189-5192 (2014).

18. Frost, J. M. *et al*. Atomistic origins of high-performance in hybrid halide perovskite solar cells. *Nano Letters* **14**, 2584-2590 (2014).
